# A multi-population multi-stage adaptive weighted large-scale multi-objective optimization algorithm framework

**DOI:** 10.1038/s41598-024-64570-y

**Published:** 2024-06-18

**Authors:** Lixue Xiong, Debao Chen, Feng Zou, Fangzhen Ge, Fuqiang Liu

**Affiliations:** 1 School of Physics and Electronic Information, Huaibei Normal University, Huaibei, 235000 China; 2 Anhui Province Key Laboratory of Intelligent Computing and Applications, Huaibei Normal University, Huaibei, 235000 China; 3 School of Computer Science and Technology, Huaibei Normal University, Huaibei, 235000 China; 4 Anhui Engineering Research Center for Intelligent Computing and Application on Cognitive Behavior (ICACB), Huaibei, 235000 China; 5 School of Electronics and Information Engineering, TongJi University, Shanghai, 200092 China; 6 School of Information Engineering, Suzhou University, Suzhou, 234000 China

**Keywords:** Applied mathematics, Computer science, Scientific data

## Abstract

Weighted optimization framework (WOF) achieves variable dimensionality reduction by grouping variables and optimizing weights, playing an important role in large-scale multi-objective optimization problems. However, because of possible problems such as duplicate weight vectors in the selection process and loss of population diversity, the algorithm is susceptible to local optimization. Therefore, this paper develops an algorithm framework called multi-population multi-stage adaptive weighted optimization (MPSOF) to improve the performance of WOF in two aspects. First, the method of using multi-population is employed to address the issue of insufficient algorithmic diversity, while simultaneously reducing the likelihood of converging towards local optima. Secondly, a processing stage is incorporated into MPSOF, where a certain number of individuals are adaptively selected for updating based on the weight information and evolutionary status of different subpopulations, targeting different types of weights. This approach alleviates the impact of repetitive weights on the diversity of newly generated individuals, avoids the drawback of easily converging to local optima when using a single type of weight for updating, and effectively balances the diversity and convergence of subpopulations. Experiments of three types designed on several typical function sets demonstrate that MPSOF exceeds the comparison algorithms in the three metrics for Inverse Generation Distance, Hypervolume and Spacing.

## Introduction

In practical applications, multi-objective optimization problems (MOPs) are widespread, such as design optimization of high-frequency transformers^1^, route planning^2^, and model training in federated learning^3^. So far, scholars have developed several effective multi-objective evolutionary algorithms (MOEAs)^4–7^ to tackle these problems. However, the search space significantly enlarges with increasing dimensions in both objective space and decision variables, leading to a severe degradation in the performance of most low-dimensional MOEAs when dealing with such problems. Therefore, LSMOPs have shown promising research prospects in recent years, and the approaches to solving LSMOPs are roughly classified into three categories.

The first type of method is optimizing the original variables of LSMOPs. These methods mainly include two types of strategies. The first strategy involves segmenting the decision variables through distinct methods, followed by separate optimization. Cooperative Coevolutionary (CC) Differential Evolution algorithm (CCGDE3)^8^ randomly partitions decision variables into equally sized subgroups, with each subgroup’s decision variables being optimized uniquely. MOEA based on random-based dynamic grouping strategy (MOEA/D-RDG)^9^ selects subgroup sizes with probability, and then randomly groups the decision variables. CC large-scale algorithm (CCLSM)^10^ groups decision variables based on a fast interdependency identification algorithm. However, the performance of these methods is closely related to the accuracy of grouping, and when the accuracy of grouping is low, it can cause a decline in the performance of the algorithm. The second strategy involves analyzing the decision variables and subsequently conducting optimization on them using different strategies for the variables of various types identified in the analysis. MOEA based on decision variable analysis (MOEA/DVA)^11^ separates the variables into mixed, distance and position variables. Taking into account population convergence, the distance variables are further subdivided into several sub-components and optimized independently. To address the difficulty in accurately classifying decision variables in MOEA/DVA, a clustering method is employed in^12^ to partition variables associated with diversity and convergence. Later, a parallel framework derived from large-scale MOEA (LMEA) is proposed in^13^, which eliminates the dependencies between sub-processes. Nonetheless, the analysis of decision variables using these methods often demands a substantial amount of computational resources.

The second type of method is the problem transformation scheme, whose main idea is using some transformation strategies to convert high-dimensional problems into low-dimensional ones. Subsequently, low-dimensional optimization algorithms are adopted along with other operators to solve LSMOPs. The characteristic of these algorithms is that they do not directly optimize the original variables of the LSMOPs. WOF^14^ achieves the transformation from the original to a weight optimization problem by assigning weights to the grouped decision variables. Since the grouping mechanism in WOF cannot provide heuristic information, a stochastic dynamic grouping approach is employed in^15^. This approach adaptively determines the group size, allowing individuals to discover more appropriate groupings during different stages of the optimization process. An adaptive variable grouping method based on Pearson correlation is developed in^16^, which adaptively divides variables as the solution evolves. To further enhance the efficiency of WOF, the two distinct optimization problems are merged into one stage in^17^. Additionally, this approach utilizes the advantages of the competitive swarm optimizer (CSO) algorithm to improve the search capability of the optimization process. However, these methods update the individuals only according to the optimal weights generated by some fixed individuals and lack guidance of the overall weight information, and the performance of these algorithms is affected to a certain extent. Large-scale multiobjective optimization framework (LSMOF)^18^ proposed a bidirectional search approach that associates the constructed motion direction vectors of partial individuals with weights, increasing the chance of intersecting the motion direction of individuals with the Pareto optimal set (PS) to improve search efficiency. However, this search direction covers a limited search space and may lead to search failure when the search direction does not point to the PS.

The third type of method is based on some novel search strategy. This method mainly generates offspring by designing a special search strategy. An efficient large-scale CSO algorithm (LMOCSO)^19^ adopts a two-pair competition mechanism to realize the learning of poor performance particles to good performance particles, and proposes an update strategy that simultaneously considers the velocity and acceleration of the particles, which effectively improves the performance of the poorer particles. However, only the poor particles are updated during each competition, the good particles are not reasonably guided, which could hinder the algorithm’s ability to break free from local optima, thereby impeding its capacity to converge towards better solutions. Inverse modeling MOEA (IM-MOEA)^20^ first transforms solutions using an inverse model that utilizes the Gaussian process as the basis from the decision space into the objective space. Subsequently, potential solutions are sampled to generate offspring through the inverse model from the objective space. However, some features of its reference vectors may pose certain challenges in handling LSMOPs. IM-MOEA based on decomposition algorithm (IM-MOEA/D)^21^ adopts a novel spatial grouping approach that employs k-means algorithm. Additionally, in order to select optimal reference vectors, it implements a selection approach using decomposition and global replacement as the basis. As this method introduces multiple parameters, adjusting different parameter values may affect its performance. Scalable small subpopulations based covariance matrix adaptation evolution strategy ($\mathrm {S^3}$-CMA-ES)^22^ adopts the variable analysis method described in the paper^11^ to divide the decision variables. Then, it applies an adaptive evolution strategy based on the covariance matrix to further divide them into smaller subgroups, and offspring are generated by iteratively updating the Gaussian model. However, for some complex problems with strong correlation between variables, the decision variables are often difficult to divide, leading to a decrease in the performance of the algorithm.

Due to the fact that WOF does not directly optimize the original variables, the algorithm’s convergence accuracy is limited to some extent. Nevertheless, it still plays a significant role in LSMOPs due to its advantages in optimizing low-dimensional variables and achieving fast convergence speeds. However, it also has some disadvantages, and there is still room for improvement in its performance. The traditional WOF employs a single-population optimisation approach, which may result in the loss of population diversity when the algorithm converges rapidly. The weight population is optimised based on selected reference solutions, and a set of weights selected from the weight population is used for population updating, which may result in the population becoming concentrated in local regions. The research found that incorporating a set of weights containing more information for population updating represents an effective method for the full utilisation of the low computational cost advantage of WOF and the avoidance of population concentration in local regions. Building upon this, a multi-population multi-stage adaptive weighted optimization algorithm framework (MPSOF) is proposed. The main contributions of MPSOF are summarized as follows.

- A three-stage algorithm framework for LSMOPs is developed. In stage I, the multiple populations strategy is implemented to maintain population diversity. In stage II, an optimization process is devised for balancing both diversity and convergence in subpopulations. In stage III, a global optimization is executed, thereby improving the distribution of solutions and algorithm convergence quality.
- An adaptive mixed-weight individual updating strategy is introduced to diversify the individual updating methods. Addressing the issues in traditional WOF where selecting optimal weights through reference directions leads to the duplication of individual update weights, reducing population diversity, and wasting computational resources, a new set of weight vectors is derived through processing the optimal weight vectors of each subpopulation during the optimization’s second stage. Based on the frequency of weight vector repetition and the evolutionary status of each subpopulation, a novel individual generation strategy is designed, which adaptively adjusts the individual updating method, balancing the exploration and exploitation capabilities of subpopulation individuals and conserving computational resources.
- To validate the efficacy of MPSOF, three types of experiments are conducted using some typical large-scale multi-objective datasets. By adopting different MOEAs within the new algorithm framework, it is proved that this framework can enhance the effectiveness of traditional MOEAs in addressing LSMOPs. Moreover, by embedding the same MOEA into different frameworks for LSMOPs, it is substantiated that the new framework has superior performance. By embedding a suboptimal algorithm into the new algorithm framework and conducting experiments on these test sets, and comparing the experimental results with those obtained from several advanced large-scale MOEAs, it is validated that MPSOF outperforms other MOEAs.

## Related work

MOPs should simultaneously consider multiple conflicting objectives during the optimization process. It can be described mathematically as:

1 $$\begin{aligned}&\min f(X)=({{f}_{1}}(X),{{f}_{2}}(X),\ldots ,{{f}_{M}}(X)) \nonumber \\&\text {subject to }X\in {{\mathbb {R}}^{d}},\text { and }{{h}_{k}}\left( X \right) \le 0,k\in \left\{ 1,2,\ldots,I \right\} \end{aligned}$$

where *f*(*X*) is an objective function with a number of *M* conflicting objectives, *X*=(${{x}_{1}}$,${{x}_{2}}$,\ldots,${{x}_{d}}$) donates the *d*-dimensional decision variable vector. *h* and *I* are constraint functions and number of constraints, respectively. $\mathbb {R}^{d}$ is feasible spaces. Due to the conflicting nature of the objective, the Pareto dominance relationship is used to distinguish the quality of two solutions. If ${{X}_{1}}$, ${{X}_{2}}$ $\in$ $\mathbb {R}^{d}$, ${{X}_{1}}$ is known as Pareto dominated ${{X}_{2}}$ (represented as ${{X}_{1}}$ $\prec$ ${{X}_{2}}$), when and only when the following conditions are met :

2 $$\begin{aligned}&\forall i\in 1,2,\ldots,M,{{f}_{i}}\left( {{X}_{1}} \right) \le {{f}_{i}}\left( {{X}_{2}} \right) \nonumber \\&\exists j\in 1,2,\ldots,M,{{f}_{j}}\left( {{X}_{1}} \right) <{{f}_{j}}\left( {{X}_{2}} \right) \end{aligned}$$

If *X* is not dominated by other solutions in $\mathbb {R}^{d}$, then *X* is called the Pareto optimal solution, the set of all Pareto optimal solutions {*X* $\in$ $\mathbb {R}^{d}$ | $\forall$ Y $\in$ $\mathbb {R}^{d}$,Y $\nprec$ X} is called the PS, {*f*(*X*) | *X*$\in$ PS} is called the Pareto front (PF). When *d* is no less than 100 and *M* is no less than 2, the optimization problems represented by Eq. (1) are often called LSMOPs.

### Basic weighted optimization framework

WOF^23,24^ contains alternating optimization and normal optimization. In alternating optimization, a transformation function is employed to convert the decision space from high-dimensional to low-dimensional based on the selected reference solution. After that, the existing MOEA searches for the optimal solution in the reduced subspace. In the normal optimization, the original variables are optimized by a selected MOEA to obtain higher quality solutions.

**Figure 1:**
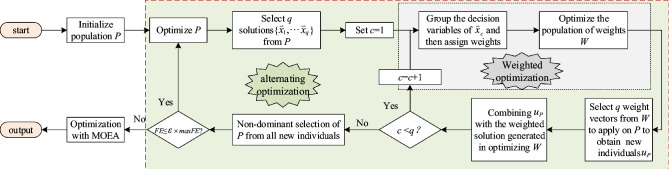
Flowchart of the WOF.

Figure 1 portrays the flowchart of the WOF. Where $\varepsilon$ is a control parameter for dividing the two stages, *FE* and *maxFE* represent the number of function evaluations at present and in total, respectively. At the alternating optimization stage, the original variables are first optimized for a predetermined number of evaluations, after which *q* reference solutions are selected using the reference direction selection strategy. For each selected solution, its decision variables are grouped and the weight population *W* is initialized. The weight population is optimized using a selected MOEA by associating with the solution. Specifically, the variables of the reference solution are grouped into $\gamma$ groups using an ordered grouping method. By associating a weight ${{w}_{j}}$ with each group, an optimization problem with a *d*-dimensional decision variable is transformed into a $\gamma$-dimensional weight optimization problem. The above transformation process can be formulated as follows.

3 $$\begin{aligned} \psi \left( \textbf{w},\textbf{x} \right) =\left( \underbrace{{{w}_{1}}{{x}_{1}},\ldots,{{w}_{1}}{{x}_{l}}}_{{{w}_{1}}},\ldots,\underbrace{{{w}_{\gamma }}{{x}_{d-l+1}},\ldots,{{w}_{\gamma }}{{x}_{d}}}_{{{w}_{\gamma }}} \right) \end{aligned}$$

The transformation function is as shown in Eq. (4)

4 $$\begin{aligned} \psi \left( {{w}_{j}},{{x}_{i}}^{\text { }\!\!'\!\!\text { }} \right) ={{x}_{i}}=\left\{ \begin{array}{*{35}{l}} {{x}_{i}}^{'}+\left( {{w}_{j}}-1.0 \right) \cdot \left( {{x}_{i,\max }}-{{x}_{i}}^{'} \right) \text { }if\text { }{{w}_{j}}>1.0 \\ {{x}_{i,\min }}+{{w}_{j}}\cdot \left( {{x}_{i}}^{'}-{{x}_{i,\min }} \right) \text { }if\text { }{{w}_{j}}\le 1.0 \\ {{w}_{j}}\in \left[ 0,2 \right] \\ \end{array} \right. \end{aligned}$$

where ${{w}_{j}}$ (*j* = 1,\ldots,$\gamma$) represents the *j*-th weight, $x_{i}^{'}$ represents the selected reference solution for optimizing the weight ${{w}_{j}}$, ${{x}_{i,max}}$ and ${{x}_{i,min}}$ are the upper and lower bounds of variable ${{x}_{i}}$ (*i* = 1,\ldots,*d*).

For the optimized weight population *W*, *q* weight individuals are selected from it and then applied to the original population to produce some new individuals. Subsequently, the newly generated individuals are merged with those obtained during the optimization process of *W*. Any duplicates are removed, and the initial population for the second stage is obtained through non-dominated selection. Finally, further optimization is performed using a suitable algorithm.

### Competitive swarm optimizer

**Figure 2:**
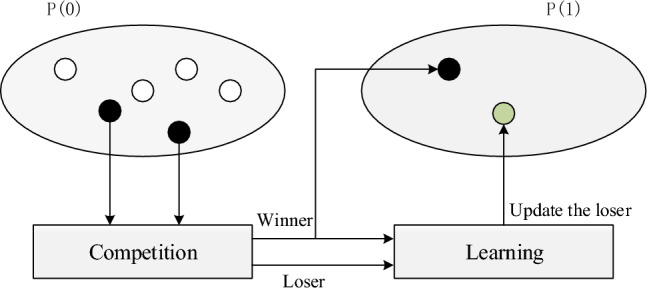
The competitive process of CSO.

The CSO algorithm^25^ takes inspiration from the collective behavior observed in biological populations, such as the particle swarm optimization (PSO) algorithm. Unlike PSO, which uses global and individual best positions to drive population evolution, CSO incorporates competition among particles to guide population evolution. The competition process is depicted in Fig. 2. In the current population, competition takes place between two randomly selected particles. The particle demonstrating exhibits superior performance is called the winner, while the other is labeled as the loser. The winner advances to the subsequent iteration, and the loser updates its position with Eq. (5).

5 $$\begin{aligned} {{v}_{L}}\left( t+1 \right)&= {{c}_{1}}{{v}_{L}}\left( t \right) +{{c}_{2}}\left( {{x}_{W}}\left( t \right) -{{x}_{L}}\left( t \right) \right) +\varphi {{c}_{3}}\left( \bar{x}\left( t \right) -{{x}_{L}}\left( t \right) \right) \nonumber \\ {{x}_{L}}\left( t+1 \right)&= {{x}_{L}}\left( t \right) +{{v}_{L}}\left( t+1 \right) \end{aligned}$$

where ${{c}_{1}}$, ${{c}_{2}}$, ${{c}_{3}}$ are three random vectors in the domain [0,1], ${{v}_{L}}$ (*t*) is the velocity of the loser, ${{x}_{W}}$ (*t*) and ${{x}_{L}}$ (*t*) are the positions of the winner and loser, $\bar{x}$ (*t*) is the global average position and $\varphi$ is the control parameter.

In recent years, due to CSO’s efficient search capabilities in addressing large-scale single objective problems^26^, Tian et al. extended CSO to solve LSMOPs and proposed an efficient optimization algorithm based on CSO^19^. They designed a two-stage approach for updating individual position, greatly enhancing the search efficiency of traditional CSO algorithm. The specific update strategy for the particles is as follows.

The fitness of each particle is calculated based on^27^. Afterward, among the competing particles, those with poor fitness learn from those with good fitness and update their own positions according to Eq. (6).

6 $$\begin{gathered} \vec{v}_{L} \left( {t + 1} \right) = c_{0} \vec{v}_{L} \left( t \right) + c_{1} \left( {\vec{x}_{W} \left( t \right) - \vec{x}_{L} \left( t \right)} \right) \hfill \\ \vec{x}_{L} \left( {t + 1} \right) = \vec{x}_{L} \left( t \right) + \vec{v}_{L} \left( {t + 1} \right) + c_{0} \left( {\vec{v}_{L} \left( {t + 1} \right) - \vec{v}_{L} \left( t \right)} \right) \hfill \\ \end{gathered}$$

where ${x}_{W}$ and ${x}_{L}$ are the positions of the winner and loser, ${c}_{0}$ and ${c}_{1}$ are random numbers between 0 and 1.

### Research motivations

Reducing the dimensionality of decision space is an effective method in addressing LSMOPs. As a classical dimensionality reduction method for LSMOPs, WOF adopts the variable grouping weighted transformation method to decrease the dimensionality of the optimization variables, thereby reducing the algorithm’s computational complexity and enhancing its convergence speed. However, the traditional WOF uses the same weights for each group of variables, and scales the variables in the same group to the same degree, which leads to difficulties in maintaining population diversity. By dynamically adjusting the evaluation numbers of the weights to change their diversity^17^, the population convergence and diversity are well balanced. However, within the same evolutionary generation, individuals still use a single set of weights to update their positions, thus limiting their ability to maintain diversity. How to design a better algorithmic framework that effectively balances population convergence and diversity is essential for enhancing algorithmic performance in LSMOPs. Under this motivation, a three-stage algorithm framework is developed. It aims to capitalize on the dimensionality reduction capability of WOF and effectively leverage its relatively small computation cost in the initial stage. Additionally, it adopts a multi-population method to sustain the population diversity and accelerate convergence speed. In the second stage, an adaptive multi-population hybrid weight position update method is designed for each subpopulation, which uses global weight information to enrich the individual weight updating method, and the convergence and diversity of subpopulations is better balanced. Since the optimization target during the optimization process is not the original variables, which exerts a definite impact on the algorithm’s convergence accuracy. Therefore, in the third stage, the population with original variables is optimized with an existing MOEA. Furthermore, the algorithmic framework of this paper also can be seamlessly integrated with different MOEAs.

**Figure 3:**
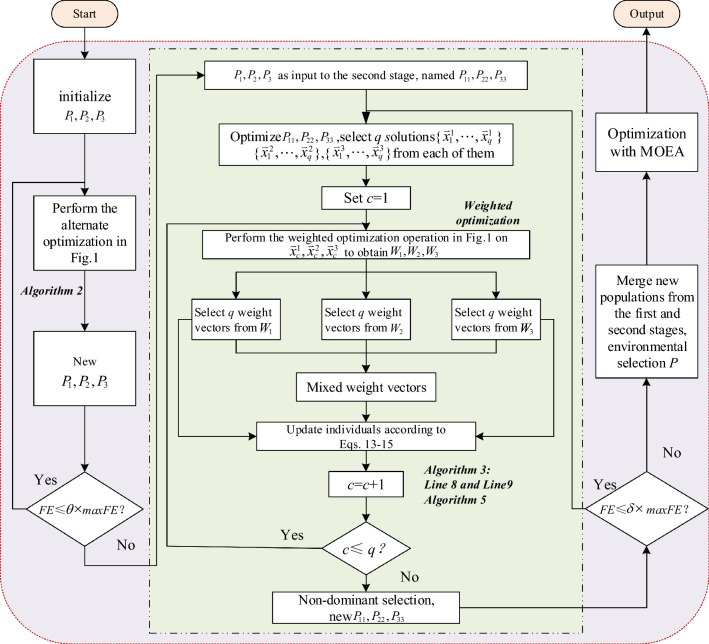
Flowchart of the MPSOF.

## The proposed MPSOF

### The framework of MPSOF

Figure 3 illustrates the basic flowchart of MPSOF. First, three populations ${{P}_{1}}$, ${{P}_{2}}$, ${{P}_{3}}$ are initialized. Then, each population adopts the same alternating optimization method as WOF in Fig. 1 for the first phase update. These updated populations are immediately used as the initial populations for the second phase, named ${{P}_{11}}$, ${{P}_{22}}$, ${{P}_{33}}$. After preliminary optimization, each of them has *q* reference solution ({$\textbf{x}_{1}^{i}$,...,$\textbf{x}_{q}^{i}$}, *i* = 1, 2, 3) selected. Then, the weighted populations are initialized and further optimized, as shown in the weighted optimization area Fig. 1. Each optimized weight population has *q* weight vectors selected. New individuals are then adaptively updated based on these weight vectors and the mixed weight vectors obtained through the fusion of these weight vectors. Finally, the initial population for the third phase is selected from the new populations generated in the first two stages. MPSOF utilizes an existing MOEA to optimize the overall variables and generate the final solution set.

Algorithm 1 presents the pseudocode of MPSOF. There are three stages in the entire algorithm. First, the initial population is divided into three subpopulations. When *FE* $\le$ $\theta$ $\times$ *maxFE*, each subpopulation executes Algorithm 2 to complete the position updating of the individual in the first stage. At this stage, the evaluations number for each optimized subpopulation is ${{t}_{1}}$, and the evaluations number for each optimized weight is ${{t}_{2}}$. The main goal of this part is to leverage the multi-population method to enhance population diversity, while fully capitalizing on the advantages of WOF, including its low computational complexity and fast execution speed. When *FE* $\le$ $\delta$ $\times$ *maxFE*, it enters the second stage of optimization. The reference solutions for the three subpopulations are obtained using the same method as in Algorithm 2. Subsequently, the weights are optimized with these reference solutions. In each weighted optimization phase, the weight variables of three subpopulation are generated. Following this, an adaptive multi-population hybrid weight updating strategy is designed to produce new offspring individuals. The concrete optimization steps of this stage are illustrated in Algorithm 3, having the primary purpose of balancing convergence and diversity using the designed updating strategy. In the third stage, a large-scale MOEA is chosen to optimize the new individuals generated from the first and second stages on a global scale, further enhancing the convergence accuracy.

**Algorithm 1:**
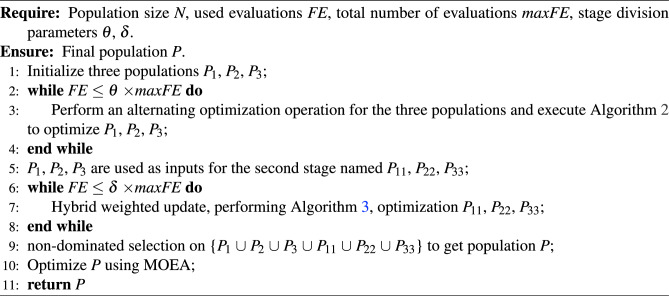
MPSOF

Algorithm 2 provides detailed steps for the alternating optimization part of MPSOF. The initial population is optimized in Line 1, and *q* solutions are chosen from each of the three population in Line 2. In the main loop (Lines 4-10), the decision variables of each selected solution are grouped and weight populations are initialized (Line 5). The weights are optimized using MOEA by combining the selected solution with the weights in the population via a transformation function (Line 6), and *q* weight vectors are selected from each of the three optimized weight populations (Line 7). These are then applied to the corresponding subpopulations to generate new individuals (Line 8). The individuals are merged in Line 9. Finally, the new population of the first stage is obtained by non-dominated sorting (Line 11).

**Algorithm 2:**
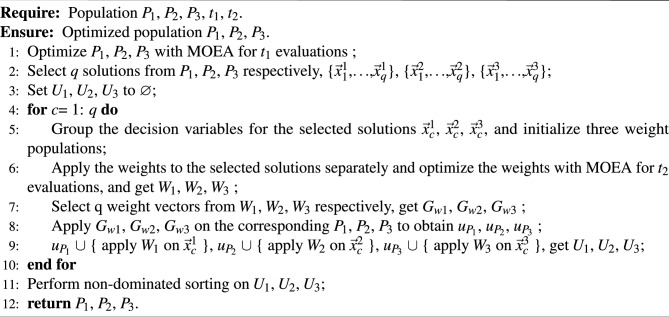
Alternating Optimization Operations

Algorithm 3 gives the specific steps for hybrid weighted updating the positions of individuals. Among them, the front 6 lines are similar to the execution process in Algorithm 2. In the main loop (Lines 4–10), the mixed weighted updating proportion factor *xr* is calculated (Line 7). Subsequently, the adaptive updating strategy is used to generate the positions of the individuals in different subpopulations (Line 8), balancing their convergence and diversity. Detailed descriptions of the hybrid proportionality factor and the adaptive multi-population hybrid weighted updating strategy will be presented in Sections “Design the mixed proportion factor” and “Adaptive multi-population mixed weights individual updating strategy”.

### Design the mixed proportion factor

In WOF, the optimal weights are obtained by reference directions^24^, the excellent weights might be selected multiple times. When the same weights are applied to the same individual, more duplicate individuals will be generated, and the algorithm will waste more computation resources on evaluating these duplicate individuals. To avoid generating many duplicate weights, a deletion strategy is adopted for multiple duplicate weights during the second stage of the algorithm. Only the weight vectors that are not duplicated among them are retained to act on the individuals. In addition, according to the proportion of repeated weights, the mixed proportion factor is designed. After deleting the duplicate weights, a specific portion of individuals are chosen to update their positions using the mixed weights, while another part of individuals update their positions using the weights of the subpopulation itself. This operator enables a trade-off in convergence and diversity. Concrete design of the mixing scale factors is presented below.

Supposing there are three weight populations, denoted as ${{W}_{1}}$, ${{W}_{2}}$, ${{W}_{3}}$, and the size of them is *k*. These weight populations are then applied separately to the corresponding reference solutions for optimisation, with the objective of obtaining the optimised weight populations. Subsequently, *q* optimal weight vectors are selected from the *k* weight vectors respectively. The number of duplicate weight vectors, represented by *num*1, *num*2, *num*3, is calculated for each set. Subsequently, the overall number of duplicate weight vectors is computed using Eq. (7). The weight repetition proportion of the current population is obtained with Eq. (8). The mixed weight contains global weight information rather than the only optimal weight of the subpopulation. Each subpopulation uses two types of weights to generate new individuals according to a designed proportion. When a large number of individuals utilize mixed weights to produce new individuals, the diversity of the subpopulation experiences significant enhancement, while its convergence is weakened. On the contrary, the convergence of the subpopulation undergoes substantial improvement, but its diversity is weakened. To balance the effect of the two types of weights, a mixed proportion factor *xr* based on *r* is designed and the value of it is limited between approximately 0.3 and 0.8 as shown in Eq. (9).

7 $$\begin{aligned}&num=num1+num2+num3 \end{aligned}$$

8 $$\begin{aligned}&r=\frac{num}{3\times q} \end{aligned}$$

9 $$\begin{aligned}&xr={{e}^{r-1.25}} \end{aligned}$$

**Algorithm 3:**
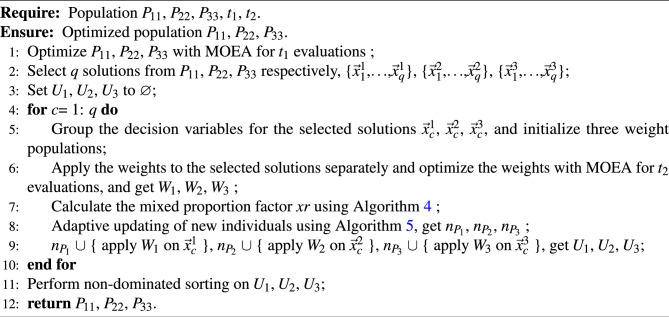
Mixed weighted updating the positions of individuals

The mixed proportion factor *xr* reflects the evolutionary state of the current population. A higher *xr* value corresponds to a larger number of the current duplicate weights, resulting in fewer non-duplicate weights being utilized to update the population. This means that the new individuals will be inclined towards local optimality, leading the population into an exploitation phase. On the contrary, the more non-repetitive weights are utilized to obtain new individuals, the individual diversity is improved, making it more likely for the population to enter the exploratory phase. When the population tends to the exploitation stage, more individuals are updated using the mixing weights to elevate the diversity and bolster the exploratory capabilities of the individuals. Conversely, when the population tends to the exploration stage, more individuals are updated using their optimal weights to enhance the population convergence. Through this operation, the exploitation and exploration capabilities of the subpopulations are balanced well. The process of determining the mixing proportion factor is shown in Algorithm 4.

**Algorithm 4:**
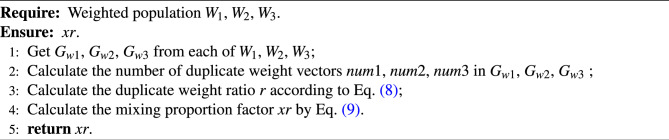
Mixing proportion factor

### Adaptive multi-population mixed weights individual updating strategy

The WOF provides an effective optimization method for LSMOPs, and also offers a new approach for using low-dimensional MOEAs to solve LSMOPs. Yet, traditional weighted optimization algorithms rely solely on a set of optimized weight vectors to update individuals. Although this approach does enhance the algorithm’s convergence speed to a certain extent, its lack of diversity may result in local optima. To fully leverage the advantages of this approach, this paper develops an adaptive multi-population hybrid weight individual updating method. The method addresses the dual challenges of enhancing population diversity and balancing subpopulation convergence with diversity. The hybrid weight individual updating method is described as follows.

For the three subpopulations in the second stage, the optimal weight vectors ${{G}_{w1}}$, ${{G}_{w2}}$, ${{G}_{w3}}$ of each subpopulation can be determined using the same method as in the WOF. Then, the weights ${{W}_{s1}}$, ${{W}_{s2}}$, ${{W}_{s3}}$ of these three subpopulations are combined to obtain weight vectors with global information. As shown in Eqs. (10) and (11), the optimal weights of three subpopulations can be different. For simplicity in this paper, the weight of three subpopulations is 1/3. Subsequently, according to the mixed ratio factor, different numbers of individuals are selected and updated using the unique optimal weight vectors of the subpopulations themselves and the mixed weight vectors, generating some initial individuals for the third stage, as shown in Eqs. (13), (14), and (15).

10 $$\begin{aligned}&G{{m}_{w}}={{W}_{s1}}\times {{G}_{w1}}+{{W}_{s2}}\times {{G}_{w2}}+{{W}_{s3}}\times {{G}_{w3}} \end{aligned}$$

11 $$\begin{aligned}&{{W}_{s1}}+{{W}_{s2}}+{{W}_{s3}}=1 \end{aligned}$$

12 $$\begin{aligned}&G{{m}_{w}}=1/3\times ({{G}_{w1}}+{{G}_{w2}}+{{G}_{w3}}) \end{aligned}$$

13 $$\begin{aligned} {{n}_{{{P}_{11}}}}&= \psi (G{{m}_{w}},xr\times {{P}_{11}}) \nonumber \\ {{n}_{{{P}_{21}}}}&= \psi (G{{m}_{w}},xr\times {{P}_{22}}) \nonumber \\ {{n}_{{{P}_{31}}}}&= \psi (G{{m}_{w}},xr\times {{P}_{33}}) \end{aligned}$$

14 $$\begin{aligned}&{{n}_{{{P}_{12}}}}=\psi ({{G}_{w1}},\left( 1-xr \right) \times {{P}_{11}}) \nonumber \\&{{n}_{{{P}_{22}}}}=\psi ({{G}_{w2}},\left( 1-xr \right) \times {{P}_{22}}) \nonumber \\&{{n}_{{{P}_{32}}}}=\psi ({{G}_{w3}},\left( 1-xr \right) \times {{P}_{33}}) \end{aligned}$$

15 $$\begin{aligned} {{n}_{{{P}_{1}}}}&= {{n}_{{{P}_{11}}}}\cup {{n}_{{{P}_{12}}}} \nonumber \\ {{n}_{{{P}_{2}}}}&= {{n}_{{{P}_{21}}}}\cup {{n}_{{{P}_{22}}}} \nonumber \\ {{n}_{{{P}_{3}}}}&= {{n}_{{{P}_{31}}}}\cup {{n}_{{{P}_{32}}}} \end{aligned}$$

where ${{G}_{w1}}$, ${{G}_{w2}}$, ${{G}_{w3}}$ denote the three sets of optimal weights selected from the three optimized weight populations, ${{Gm}_{w}}$ is a set of weights obtained by fusing the weights of multiple subpopulations and $\psi$ is the transformation function. Algorithm 5 shows the pseudocode for the individual updating process.

**Algorithm 5:**
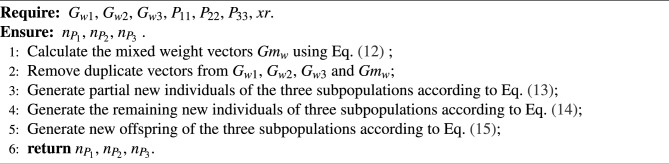
Adaptive update strategy

## Experimental studies

In this section, three types of experiments are designed to validate the effectiveness of the developed algorithm framework (MPSOF) from various perspectives. The first type of experiment involves using different optimization algorithms in the new framework to assess its impact on the original algorithm performance. The second type of experiments involves applying the fast and elitist genetic algorithm (NSGAII)^28^ to three different optimization frameworks, WOF^24^, LSMOF^18^ and MPSOF for evaluating the performance of these frameworks. The third category of experiment involves embedding the general performance algorithm LMOCSO into MPSOF, and then comparing it with seven advanced large-scale MOEAs using the LSMOP, WFG, and UF test function problems.

### Quality metrics

In experiments, the evaluation of the algorithms’ performance relies on commonly used performance indicators, Inverse Generation Distance (IGD)^29^, Hypervolume (HV)^30^ and Spacing (SP)^31^.

IGD is defined by Eq. (16). Where *S* symbolizes the true PS, *P* represents the approximate PS, *N* denotes the population size, and *dis*(${{p}_{i}}$, *S*) signifies the minimum Euclidean distance between points ${{p}_{i}}$ and *S*. IGD quantifies the algorithm diversity and convergence by computing the mean distances from the closest generated solutions to the true Pareto front (PF). A lower IGD value signifies that the algorithm has superior comprehensive performance.

16 $$\begin{aligned} IGD(S,P)=\frac{\sum \limits _{i=1}^{N}{{{p}_{i}}\in P\text { dis(}{{p}_{i}},S\text {)}}}{N} \end{aligned}$$

HV is defined as shown in Eq. (17), which measures the algorithm performance by quantifying the hypervolume formed between the generated solutions and the reference points. It is an effective one-dimensional quality metric that is strictly monotonic in terms of Pareto domination. A higher value of HV suggests that the algorithm possesses a greater capability to cover the real PF. Here, *P* represents the approximate optimal solutions achieved by the algorithm in objective space, ${{a}^{i}}$ = ${{\text {(}a_{1}^{i},a_{2}^{i},\ldots ,a_{m}^{i})}^{T}}$ is the reference point for selection, ${{D}_{0}}$(.) denotes the Lebesgue measure.

17 $$\begin{aligned} HV={{D}_{0}}\left( \bigcup \limits _{{{p}_{j}}\in P}{\left[ {{f}_{1}}(x),a_{1}^{i} \right] }\times \cdots \times \left[ {{f}_{m}}(x),a_{m}^{i} \right] \right) \end{aligned}$$

SP is defined by Eq. (18), which represents the distribution of individuals within the approximate PS in target space. It is a diversity evaluation indicator. A lower SP value corresponds to a more evenly distributed solution set. Here, *N* is the population size, ${{Dis}_{q}}$ is the Euclidean distance between the *q*-th and the nearest individuals, $\bar{Dis}$ is the average distance.

18 $$\begin{aligned} SP = \frac{{{{\left[ {\frac{1}{N}\sum \limits _{q = 1}^N {{{\left( {Di{s_q} - \bar{Dis} } \right) }^2}} } \right] }^{\frac{1}{2}}}}}{{\bar{Dis} }} \end{aligned}$$

### Experimental parameters

#### Experimental settings

In this paper, each algorithm is performed independently 20 times on the PlatEMO^32^ platform, with *maxFE* set to 100000 as the termination criterion and *N* set to 105. The two main parameters in MPSOF are obtained by parameter sensitivity analysis: $\theta$ is 0.4, $\delta$ is 0.6, while the other parameters remain consistent with those of WOF. Among them, ${{t}_{1}}$ is 1000, ${{t}_{2}}$ is 500, $\gamma$ is 4, the initialized weight population size *k* equals 10, the number of selected weight vectors and reference solutions *q* equals *M*+1. Other comparison algorithms adopt the advised parameter settings provided in the relevant papers. Statistical results are obtained through the utilization of Wilcoxon rank-sum test with a significance level of 0.05. The bold font indicates the optimal solutions, and the symbols “+/−/=” represent whether the comparison algorithms are considerably superior, inferior, or similar to MPSOF.

**Table 1:** Average IGD values for LSMOP1, LSMOP5 and LSMOP8 with $\delta$ change from 0.1 to 0.9 under the interval 0.1 when $\theta$ is different.

Parameter	$\theta$	0.1	0.2	0.3	0.4	0.5	0.6	0.7	0.8	0.9
Problem	LSMOP1	0.2196	0.1948	0.1852	**0.1681**	0.1748	0.1707	0.1714	0.1785	0.2086
	LSMOP5	0.3361	**0.3360**	0.3427	0.3389	0.3487	0.3367	0.3559	0.3460	0.3991
	LSMOP8	0.0786	0.0792	0.0747	0.0737	0.0743	**0.0736**	0.0738	0.0749	0.0828
Average IGD		0.2114	0.2033	0.2009	**0.1936**	0.1993	0.1937	0.2004	0.1998	0.2302

#### Parameter sensitivity analysis

$\theta$ and $\delta$ are two important parameters of MPSOF, which divide the whole algorithm into three stages. Different parameters will have different impacts on the algorithm’s performance. To determine these two parameters, parameter sensitivity analysis is used in this paper. Three functions with different properties were selected from LSMOP for the experiments, where the decision variable dimension and the number of objectives are respectively 500 and 3. $\theta$ and $\delta$ change from 0.1 to 0.9 respectively with an interval of 0.1. The first experiment is the average IGD value obtained by the three functions as $\delta$ takes different values and $\theta$ changes from 0.1 to 0.9 ($\theta$ $\le$ $\delta$). The results are shown in Table 1. For example, “0.1681” in Table 1 indicates that the average IGD is 0.1681 with $\delta$ change from 0.4 to 0.9 under the interval 0.1 when $\theta$ is 0.4 for LSMOP1. The second experiment is the average IGD value obtained by the three functions as $\theta$ takes different values and $\delta$ changes from 0.1 to 0.9 ($\theta$ $\le$ $\delta$). The results are shown in Table 2. For example, “0.0704” in Table 2 indicates that the average IGD is 0.0704 with $\theta$ change from 0.1 to 0.6 under the interval 0.1 when $\delta$ is 0.6. The last lines of the two tables are the average IGD of LSMOP1, LSMOP5 and LSMOP8.

From Table 1, the test results for the three typical functions show that the average IGD value is minimized when $\theta$ is 0.4, so $\theta$ is set to 0.4 in this paper. From Table 2, the test results for these three functions demonstrate that the average IGD value is minimized when $\delta$ is 0.6. Taking into account the average characteristics impact of parameters on the algorithm, this paper has chosen to set $\delta$ as 0.6.

**Table 2:** Average IGD values for LSMOP1, LSMOP5 and LSMOP8 with $\theta$ change from 0.1 to 0.9 under the interval 0.1 when $\delta$ is different.

Parameter	$\delta$	0.1	0.2	0.3	0.4	0.5	0.6	0.7	0.8	0.9
Problem	LSMOP1	0.3093	0.3146	0.2384	0.1986	0.1674	0.1672	**0.1608**	0.1666	0.1923
	LSMOP5	0.3130	0.3160	0.3143	**0.3116**	0.3277	0.3211	0.3412	0.3629	0.3760
	LSMOP8	0.0897	0.0772	0.0746	0.0740	0.0741	**0.0704**	0.0745	0.0731	0.0847
Average IGD		0.2373	0.2359	0.2091	0.1947	0.1897	**0.1862**	0.1922	0.2009	0.2177

### Experimental results and analysis

In experiments, the algorithms’ performance in solving LSMOPs is evaluated by testing 2-objective and 3-objective problems with variables of 500 and 1000 dimensions.

#### General performance of MPSOF

The main intention of the experiments in this part is to validate that MPSOF has an enhancing effect on the performance of different embedded algorithms. Three different classical algorithms NSGAII^28^, a competitive PSO for multi objective (CMOPSO)^33^ and LMOCSO^19^ are applied in MPSOF. Tables 3 and 4 display the statistical data of IGD and HV values obtained from LSMOP by different algorithms.

Table 3 shows that MPSNSGAII is significantly superior to NSGAII in 32 test instances out of 36 test instances. Similarly, MPSCMOPSO outperforms CMOPSO in 34 test instances. Compared to the original algorithm, MPSNSGAII and MPSCMOPSO exhibit slightly lower performance on LSMOP2 with 3-objective, but the difference between them is minimal. This is mainly because the classical algorithm itself has good performance when dealing with simpler linear problems, and maintaining population diversity does not significantly increase the accuracy of the performance of the algorithm. MPSLMOCSO demonstrates superior performance over LMOCSO in 33 out of 36 test instances. For the LSMOP9 with three objectives of discontinuous PF, MPSLMOCSO performs worse than LMOCSO. According to the results from the final row of tests, NSGAII, CMOPSO, and LMOCSO outperform the algorithm in this paper on 3, 2, and 3 test instances, are inferior to the algorithm in this paper on 32, 34, and 33 test instances, and are similar in performance to the algorithm in this paper on 1, 0, and 0 test instances. This indicates that MPSOF is capable of effectively utilizing existing MOEAs to tackle LSMOPs, as the complexity of the optimization problem has been significantly reduced.

**Table 3:** The statistical IGD results with NSGAII, MPSNSGAII, CMOPSO, MPSCMOPSO, LMOCSO, and MPSLMOCSO on 36 LSMOP test problems.

Problem	M	D	NSGAII	MPSNSGAII	CMOPSO	MPSCMOPSO	LMOCSO	MPSLMOCSO
LSMOP1	2	500	1.7489e+0 (2.05e−1) −	**2.7998e−1 (7.08e−2)**	7.7653e−1 (1.92e−1) −	**2.7972e−1 (6.47e−2)**	5.9190e−1 (6.60e−2) −	**8.2846e−2 (4.79e−2)**
		1000	3.4994e+0 (1.91e−1) −	**2.9102e−1 (6.96e−2)**	1.9627e+0 (1.47e−1) −	**2.5363e−1 (4.33e−2**	1.4916e+0 (1.56e−1) −	**8.1982e−2 (3.19e−2)**
	3	500	3.8275e+0 (4.60e−1) −	**4.3586e−1 (5.64e−2)**	2.9814e+0 (5.81e−1) −	**4.6541e−1 (4.41e−2)**	1.3893e+0 (1.00e−1) −	**1.5729e−1 (4.84e−2)**
		1000	6.0673e+0 (6.55e−1) −	**4.5569e−1 (7.75e−2)**	5.8058e+0 (5.81e−1) −	**4.7055e−1 (4.95e−2)**	1.6689e+0 (9.42e−2) −	**1.3813e−1 (2.67e−2)**
LSMOP2	2	500	5.9400e−2 (1.35e−3) −	**2.3128e−2 (2.40e−3)**	5.4794e−2 (1.14e−3) −	**2.5962e−2 (1.47e−3)**	4.4205e−2 (7.24e−4) −	**5.6351e−3 (2.48e−4)**
		1000	3.6161e−2 (3.78e−4) −	**1.7695e−2 (7.15e−4)**	3.6976e−2 (7.90e−4) −	**1.6793e−2 (3.06e−4)**	2.4728e−2 (5.42e−4) −	**3.5193e−3 (1.29e−4)**
	3	500	**8.3519e−2 (4.12e−3) +**	8.6782e−2 (3.97e−3)	**6.9317e−2 (3.65e−4) +**	6.9827e−2 (4.41e−4)	5.6939e−2 (4.77e−4) −	**5.1420e−2 (1.57e−3)**
		1000	**6.6451e−2 (3.03e−3) =**	6.9040e−2 (4.13e−3)	**5.1897e−2 (4.14e−4) +**	5.2333e−2 (5.62e−4)	4.6715e−2 (2.83e−4) −	**2.6768e−2 (5.11e−4)**
LSMOP3	2	500	1.7414e+1 (2.17e+0) −	**1.4361e+0 (1.76e−1)**	2.2627e+1 (4.31e+0) −	**1.2748e+0 (8.82e−2)**	**9.6407e−1 (3.70e−1) +**	1.2556e+0 (1.10e−1)
		1000	2.0806e+1 (1.13e+0) −	**1.5758e+0 (8.10e−4)**	3.0166e+1 (1.17e+0) −	**1.5027e+0 (5.09e−2)**	2.7281e+0 (8.74e−1) −	**1.1831e+0 (2.94e−1)**
	3	500	1.3194e+1 (2.35e+0) −	**8.6071e−1 (2.95e−5)**	1.1955e+1 (5.53e−1) −	**8.6072e−1 (7.32e−15)**	1.2256e+1 (3.27e+0) −	**8.2089e−1 (2.08e−2)**
		1000	1.9211e+1 (3.99e+0) −	**8.5837e−1 (1.05e−2)**	1.4710e+1 (7.74e−1) −	**8.7561e−1 (6.66e−2)**	1.2815e+1 (2.38e+0) −	**8.5945e−1 (3.46e−3)**
LSMOP4	2	500	9.1375e−2 (2.11e−3) −	**5.1799e−2 (1.99e−3)**	8.3928e−2 (2.09e−3) −	**5.6181e−2 (1.63e−3)**	8.6265e−2 (1.25e−3) −	**3.2578e−2 (6.41e−4)**
		1000	6.0234e−2 (9.83e−4) −	**3.2524e−2 (1.65e−3)**	6.5509e−2 (1.23e−3) −	**3.4909e−2 (1.57e−3)**	5.1730e−2 (5.02e−4) −	**1.7460e−2 (3.01e−4)**
	3	500	**1.9229e−1 (6.23e−3) +**	2.1081e−1 (8.33e−3)	2.0083e−1 (2.35e−3) −	**1.7566e−1 (4.00e−3)**	1.5238e−1 (1.69e−3) −	**9.0645e−2 (3.59e−3)**
		1000	**1.2670e−1 (3.93e−3) +**	1.4053e−1 (4.84e−3)	1.2137e−1 (1.13e−3) −	**1.1079e−1 (2.38e−3)**	9.6147e−2 (9.85e−4) −	**5.2447e−2 (1.33e−3)**
LSMOP5	2	500	5.2049e+0 (5.59e−1) −	**4.3597e−1 (4.61e−2)**	2.6423e+0 (4.16e−1) −	**4.2434e−1 (4.82e−2)**	1.2830e+0 (1.72e−1) −	**2.3576e−1 (6.89e−2)**
		1000	1.0254e+1 (7.25e−1) −	**5.6179e−1 (3.11e−2)**	6.4081e+0 (3.32e−1) −	**6.7927e−1 (2.22e−2)**	3.5842e+0 (3.76e−1) −	**2.7719e−1 (1.22e−1)**
	3	500	9.7203e+0 (7.11e−1) −	**5.0504e−1 (1.82e−2)**	1.0066e+1 (7.15e−1) −	**5.0405e−1 (2.96e−2)**	2.8109e+0 (2.62e−1) −	**3.0377e−1 (6.78e−2)**
		1000	1.4748e+1 (7.48e−1) −	**4.8963e−1 (4.16e−2)**	1.5666e+1 (2.60e+0) −	**5.1359e−1 (8.67e−3)**	3.6404e+0 (2.78e−1) −	**2.9252e−1 (1.26e−1)**
LSMOP6	2	500	8.0798e−1 (1.94e−3) −	**4.2103e−1 (4.47e−2)**	7.6432e−1 (9.84e−2) −	**4.6702e−1 (2.36e−1)**	7.7482e−1 (3.73e−2) −	**3.8639e−1 (1.59e−1)**
		1000	7.7455e−1 (5.29e−4) −	**4.3718e−1 (8.52e−2)**	6.6801e−1 (1.33e−1) −	**4.5718e−1 (2.49e−1)**	7.6809e−1 (3.29e−3) −	**3.5678e−1 (1.20e−1)**
	3	500	1.6940e+3 (1.21e+3) −	**1.2853e+0 (1.12e−3)**	2.9861e+2 (3.28e+2) −	**1.3263e+0 (1.14e−1)**	1.1433e+2 (1.12e+2) −	**9.4839e−1 (2.55e−1)**
		1000	9.5597e+3 (2.08e+3) −	**1.3074e+0 (7.45e−4)**	4.2740e+3 (1.28e+3) −	**1.4213e+0 (1.74e−1)**	4.7222e+2 (2.59e+2) −	**8.2596e−1 (2.16e−1)**
LSMOP7	2	500	2.7102e+2 (4.66e+2) −	**1.5033e+0 (9.93e−4)**	1.2350e+1 (3.34e+1) −	**1.4993e+0 (1.84e−3)**	8.7638e+1 (1.21e+2) −	**1.4829e+0 (6.40e−2)**
		1000	2.7445e+3 (2.18e+3) −	**1.5110e+0 (5.87e−4)**	6.2170e+2 (7.08e+2) −	**1.5091e+0 (7.99e−4)**	1.8538e+3 (9.73e+2) −	**1.4780e+0 (7.02e−2)**
	3	500	1.3066e+0 (9.87e−3) −	**8.7012e−1 (2.82e−3)**	1.3303e+0 (1.21e−2) −	**8.8264e−1 (1.35e−2)**	9.8316e−1 (6.11e−2) −	**8.4199e−1 (5.84e−2)**
		1000	1.1079e+0 (3.04e−3) −	**8.5039e−1 (9.58e−4)**	6.9155e+2 (3.09e+3) −	**8.5701e−1 (2.07e−2)**	9.8640e−1 (3.96e−2) −	**7.9596e−1 (1.02e−1)**
LSMOP8	2	500	1.5731e+0 (1.84e−1) −	**5.7144e−1 (1.05e−1)**	1.4649e+0 (4.17e−1) −	**4.2686e−1 (2.59e−2)**	1.2412e+0 (1.55e−1) −	**1.3635e−1 (4.62e−2)**
		1000	4.7934e+0 (4.38e−1) −	**6.6607e−1 (1.78e−1)**	3.6783e+0 (2.56e−1) −	**6.3386e−1 (2.97e−2)**	2.7801e+0 (3.55e−1) −	**1.6998e−1 (1.28e−1)**
	3	500	9.4584e−1 (5.42e−2) −	**2.3200e−1 (3.31e−2)**	5.6024e−1 (4.60e−2) −	**2.0598e−1 (1.98e−2)**	6.4689e−1 (1.44e−1) −	**6.9156e−2 (7.65e−3)**
		1000	9.3512e−1 (4.52e−2) −	**2.2963e−1 (2.93e−2)**	9.3434e−1 (4.32e−2) −	**2.0085e−1 (1.18e−2)**	6.9093e−1 (1.70e−1) −	**4.6251e−2 (1.03e−2)**
LSMOP9	2	500	1.2369e+0 (8.46e−3) −	**8.0848e−1 (1.03e−3)**	1.2208e+0 (8.39e−3) −	**8.0642e−1 (2.60e−3)**	5.1330e−1 (6.42e−2) −	**1.4863e−1 (1.81e−1)**
		1000	1.3479e+0 (1.34e−1) −	**8.0426e−1 (2.61e−3)**	1.0251e+0 (1.86e−3) −	**7.9406e−1 (1.31e−2)**	1.1016e+0 (2.38e−1) −	**1.6827e−1 (2.10e−1)**
	3	500	3.5589e+0 (3.90e−1) −	**1.1647e+0 (8.78e−2)**	1.3335e+0 (3.78e−2) −	**1.1468e+0 (7.22e−4)**	**7.8551e−1 (1.17e−1) +**	1.1470e+0 (1.35e−3)
		1000	1.3180e+1 (1.58e+0) −	**1.1450e+0 (2.84e−4)**	6.1218e+0 (1.69e+0) −	**1.1470e+0 (8.93e−4)**	**7.7224e−1 (1.14e−1) +**	1.1441e+0 (4.83e−3)
+/−/=			3/32/1		2/34/0		3/33/0

**Table 4:** The statistical HV results with NSGAII, MPSNSGAII, CMOPSO, MPSCMOPSO, LMOCSO, and MPSLMOCSO on 36 LSMOP test problems.

Problem	M	D	NSGAII	MPSNSGAII	CMOPSO	MPSCMOPSO	LMOCSO	MPSLMOCSO
LSMOP1	2	500	0.0000e+0 (0.00e+0) −	**2.0263e−1 (9.02e−2)**	2.1073e−3 (4.39e−3) −	**2.1075e−1 (5.64e−2)**	7.5084e−2 (3.06e−2) −	**4.8366e−1 (5.52e−2)**
		1000	0.0000e+0 (0.00e+0) −	**2.1012e−1 (8.25e−2)**	0.0000e+0 (0.00e+0) −	**2.1422e−1 (3.95e−2)**	0.0000e+0 (0.00e+0) −	**4.8590e−1 (3.50e−2)**
	3	500	0.0000e+0 (0.00e+0) −	**3.1348e−1 (6.88e−2)**	0.0000e+0 (0.00e+0) −	**2.5874e−1 (4.90e−2)**	0.0000e+0 (0.00e+0) −	**7.1462e−1 (2.20e−2)**
		1000	0.0000e+0 (0.00e+0) −	**2.8984e−1 (8.50e−2)**	0.0000e+0 (0.00e+0) −	**2.5874e−1 (4.90e−2)**	0.0000e+0 (0.00e+0) −	**6.9746e−1 (4.52e−2)**
LSMOP2	2	500	5.0724e−1 (1.68e−3) −	**5.5430e−1 (2.94e−3)**	5.1393e−1 (1.46e−3) −	**5.5221e−1 (1.75e−3)**	5.2713e−1 (9.35e−4) −	**5.7934e−1 (3.08e−4)**
		1000	5.3700e−1 (5.57e−4) −	**5.6178e−1 (8.79e−4)**	5.3679e−1 (1.00e−3) −	**5.6364e−1 (3.70e−4)**	5.5258e−1 (7.76e−4) −	**5.8194e−1 (1.56e−4)**
	3	500	**7.7610e−1 (3.68e−3) =**	7.7421e−1 (3.87e−3)	**7.9692e−1 (3.79e−4) +**	7.9646e−1 (5.72e−4)	8.1053e−1 (7.19e−4) −	**8.3751e−1 (1.52e−3)**
		1000	**8.0022e−1 (3.31e−3) +**	7.9680e−1 (3.60e−3)	**7.9692e−1 (3.79e−4) +**	7.9646e−1 (5.72e−4)	8.2519e−1 (4.46e−4) −	**8.4744e−1 (5.49e−4)**
LSMOP3	2	500	0.0000e+0 (0.00e+0) =	0.0000e+0 (0.00e+0)	0.0000e+0 (0.00e+0) =	0.0000e+0 (0.00e+0)	**2.2180e−4 (9.92e−4) =**	0.0000e+0 (0.00e+0)
		1000	0.0000e+0 (0.00e+0) =	0.0000e+0 (0.00e+0)	0.0000e+0 (0.00e+0) =	0.0000e+0 (0.00e+0)	0.0000e+0 (0.00e+0) =	0.0000e+0 (0.00e+0)
	3	500	0.0000e+0 (0.00e+0) −	**9.0913e−2 (6.84e−6)**	0.0000e+0 (0.00e+0) −	**9.0909e−2 (8.24e−15)**	0.0000e+0 (0.00e+0) −	**9.0910e−2 (3.37e−6)**
		1000	0.0000e+0 (0.00e+0) −	**9.0914e−2 (7.64e−6)**	0.0000e+0 (0.00e+0) −	**9.0909e−2 (8.24e−15)**	0.0000e+0 (0.00e+0) −	**9.0909e−2 (1.84e−7)**
LSMOP4	2	500	4.6834e−1 (2.38e−3) −	**5.1533e−1 (2.53e−3)**	4.7818e−1 (2.43e−3) −	**5.1215e−1 (1.98e−3)**	4.7629e−1 (1.42e−3) −	**5.4484e−1 (7.66e−4)**
		1000	5.0663e−1 (1.20e−3) −	**5.4159e−1 (2.01e−3)**	5.0120e−1 (1.45e−3) −	**5.4028e−1 (1.90e−3)**	5.1737e−1 (6.11e−4) −	**5.6474e−1 (3.71e−4)**
	3	500	**6.2560e−1 (8.52e−3) +**	6.1506e−1 (1.04e−2)	6.3270e−1 (3.09e−3) −	**6.7503e−1 (4.82e−3)**	6.8678e−1 (2.29e−3) −	**7.9382e−1 (2.18e−3)**
		1000	**7.1521e−1 (8.41e−3) +**	7.0717e−1 (4.98e−3)	6.3270e−1 (3.09e−3) −	**6.7503e−1 (4.82e−3)**	7.5948e−1 (1.26e−3) −	**8.2206e−1 (1.33e−3)**
LSMOP5	2	500	0.0000e+0 (0.00e+0) −	**9.1991e−2 (4.84e−3)**	0.0000e+0 (0.00e+0) −	**9.0909e−2 (4.27e−17)**	0.0000e+0 (0.00e+0) −	**1.4353e−1 (4.50e−2)**
		1000	0.0000e+0 (0.00e+0) −	**9.0909e−2 (4.27e−17)**	0.0000e+0 (0.00e+0) −	**9.0909e−2 (4.27e−17)**	0.0000e+0 (0.00e+0) −	**1.3981e−1 (5.42e−2)**
	3	500	0.0000e+0 (0.00e+0) −	**3.4458e−1 (5.52e−4)**	0.0000e+0 (0.00e+0) −	**3.4206e−1 (2.46e−3)**	0.0000e+0 (0.00e+0) −	**3.6789e−1 (3.77e−2)**
		1000	0.0000e+0 (0.00e+0) −	**3.4482e−1 (3.64e−4)**	0.0000e+0 (0.00e+0) −	**3.4206e−1 (2.46e−3)**	0.0000e+0 (0.00e+0) −	**3.7310e−1 (4.11e−2)**
LSMOP6	2	500	0.0000e+0 (0.00e+0) −	**6.9013e−2 (2.74e−3)**	1.0101e−3 (1.61e−3) −	**6.3853e−2 (1.55e−2)**	1.5362e−2 (8.28e−3) −	**9.3337e−2 (2.03e−2)**
		1000	2.8712e−2 (8.82e−4) −	**8.0290e−2 (2.04e−3)**	3.5682e−2 (3.52e−3) −	**7.4938e−2 (1.77e−2)**	3.9205e−2 (5.12e−3) −	**1.0453e−1 (1.57e−2)**
	3	500	0.0000e+0 (0.00e+0) =	0.0000e+0 (0.00e+0)	0.0000e+0 (0.00e+0) =	0.0000e+0 (0.00e+0)	0.0000e+0 (0.00e+0) −	**8.7546e−2 (4.84e−2)**
		1000	0.0000e+0 (0.00e+0) =	0.0000e+0 (0.00e+0)	0.0000e+0 (0.00e+0) =	0.0000e+0 (0.00e+0)	0.0000e+0 (0.00e+0) −	**7.0928e−2 (5.49e−2)**
LSMOP7	2	500	0.0000e+0 (0.00e+0) =	0.0000e+0 (0.00e+0)	0.0000e+0 (0.00e+0) =	0.0000e+0 (0.00e+0)	0.0000e+0 (0.00e+0) =	0.0000e+0 (0.00e+0)
		1000	0.0000e+0 (0.00e+0) =	0.0000e+0 (0.00e+0)	0.0000e+0 (0.00e+0) =	0.0000e+0 (0.00e+0)	0.0000e+0 (0.00e+0) =	0.0000e+0 (0.00e+0)
	3	500	0.0000e+0 (0.00e+0) =	0.0000e+0 (0.00e+0)	0.0000e+0 (0.00e+0) =	0.0000e+0 (0.00e+0)	**6.0897e−2 (4.12e−2) +**	1.2988e−5 (5.81e−5)
		1000	0.0000e+0 (0.00e+0) −	**8.6613e−3 (3.51e−3)**	0.0000e+0 (0.00e+0) =	0.0000e+0 (0.00e+0)	**4.1134e−2 (3.19e−2) =**	2.6521e−2 (9.42e−3)
LSMOP8	2	500	0.0000e+0 (0.00e+0) −	**9.5849e−2 (2.19e−2)**	0.0000e+0 (0.00e+0) −	**9.3099e−2 (3.53e−3)**	0.0000e+0 (0.00e+0) −	**2.1990e−1 (2.57e−2)**
		1000	0.0000e+0 (0.00e+0) −	**1.0139e−1 (3.23e−2)**	0.0000e+0 (0.00e+0) −	**9.0909e−2 (6.88e−12)**	0.0000e+0 (0.00e+0) −	**2.1246e−1 (3.26e−2)**
	3	500	5.1595e−2 (7.16e−4) −	**3.3941e−1 (3.21e−2)**	5.3520e−2 (9.50e−4) −	**3.9648e−1 (9.10e−3)**	6.9570e−2 (1.09e−2) −	**5.3277e−1 (1.84e−2)**
		1000	6.7764e−2 (5.11e−4) −	**3.4080e−1 (3.15e−2)**	5.3520e−2 (9.50e−4) −	**3.9648e−1 (9.10e−3)**	8.0420e−2 (6.36e−3) −	**5.3521e−1 (1.81e−2)**
LSMOP9	2	500	0.0000e+0 (0.00e+0) −	**9.1102e−2 (1.30e−4)**	0.0000e+0 (0.00e+0) −	**9.1363e−2 (3.44e−4)**	7.5389e−2 (1.27e−2) −	**2.0297e−1 (2.39e−2)**
		1000	0.0000e+0 (0.00e+0) −	**9.1690e−2 (3.65e−4)**	3.5599e−2 (4.81e−4) −	**9.3177e−2 (1.89e−3)**	1.0917e−2 (1.09e−2) −	**2.0697e−1 (2.95e−2)**
	3	500	0.0000e+0 (0.00e+0) −	**1.4477e−1 (1.27e−2)**	2.9574e−2 (2.34e−3) −	**1.4698e−1 (1.93e−4)**	5.5430e−2 (3.13e−3) −	**1.4736e−1 (1.56e−4)**
		1000	0.0000e+0 (0.00e+0) −	**1.4759e−1 (1.78e−5)**	2.9574e−2 (2.34e−3) −	**1.4698e−1 (1.93e−4)**	5.6537e−2 (5.04e−3) −	**1.4742e−1 (6.02e−4)**
+/−/=			3/25/8		2/26/8		1/30/5	

Table 4 displays that the MPSOF has demonstrated strong performance across the majority of the test problems (LSMOP1, LSMOP5, LSMOP8, and LSMOP9). Additionally, MPSLMOCSO only performs slightly lower than LMOCSO on the LSMOP3 with 2-objective and LSMOP7 with 3-objective. A zero value of HV means that the compared algorithm cannot generate any solutions that meet the objective requirements. The table also indicates that among 36 test instances, NSGAII, CMOPSO, and LMOCSO are superior to the MPSOF on 3, 2, and 1 instances respectively, inferior to the MPSOF on 25, 26, and 30 instances, and show similar performance to MPSOF on 8, 8, and 5 instances.

**Table 5:** The statistical IGD results with WOF-NSGAII, LS-NSGAII, and MPSNSGAII on 36 LSMOP test problems.

Problem	M	D	WOF-NSGAII	LS-NSGAII	MPSNSGAII
LSMOP1	2	500	6.0796e−1 (8.68e−2) −	5.8955e−1 (2.70e−2) −	**2.7998e−1 (7.08e−2)**
		1000	6.2950e−1 (1.03e−1) −	6.1955e−1 (1.67e−2) −	**2.9102e−1 (6.96e−2)**
	3	500	4.9781e−1 (4.34e−2) −	5.5938e−1 (6.31e−3) −	**4.3586e−1 (5.64e−2)**
		1000	5.8681e−1 (2.59e−2) −	6.0337e−1 (9.23e−3) −	**4.5569e−1 (7.75e−2)**
LSMOP2	2	500	2.8667e−2 (2.75e−3) −	2.3532e−2 (7.77e−4) =	**2.3128e−2 (2.40e−3)**
		1000	1.8867e−2 (3.95e−4) −	1.8087e−2 (5.44e−4) −	**1.7695e−2 (7.15e−4)**
	3	500	8.7423e−2 (3.20e−3) =	8.8253e−2 (2.83e−3) =	**8.6782e−2 (3.97e−3)**
		1000	6.9651e−2 (4.10e−3) =	7.1351e−2 (4.45e−3) =	**6.9040e−2 (4.13e−3)**
LSMOP3	2	500	1.4982e+0 (9.27e−2) =	1.5645e+0 (7.32e−4) −	**1.4361e+0 (1.76e−1)**
		1000	1.5800e+0 (1.99e−3) −	**1.5734e+0 (4.73e−4) +**	1.5758e+0 (8.10e−4)
	3	500	8.6320e−1 (4.38e−2) =	**8.4821e−1 (1.11e−2) +**	8.6071e−1 (2.95e−5)
		1000	8.6243e−1 (5.63e−3) −	8.6051e−1 (4.60e−4) −	**8.5837e−1 (1.05e−2)**
LSMOP4	2	500	6.5453e−2 (5.23e−3) −	**5.1326e−2 (9.36e−4) =**	5.1799e−2 (1.99e−3)
		1000	4.0956e−2 (3.35e−3) −	3.2572e−2 (1.14e−3) =	**3.2524e−2 (1.65e−3)**
	3	500	2.1572e−1 (6.31e−3) =	2.2008e−1 (5.01e−3) −	**2.1081e−1 (8.33e−3)**
		1000	1.4214e−1 (4.55e−3) =	1.4421e−1 (4.88e−3) −	**1.4053e−1 (4.84e−3)**
LSMOP5	2	500	5.6768e−1 (1.72e−1) =	7.4209e−1 (2.28e−16) −	**4.3597e−1 (4.61e−2)**
		1000	6.4234e−1 (9.23e−2) −	7.4209e−1 (2.28e−16) −	**5.6179e−1 (3.11e−2)**
	3	500	5.3303e−1 (2.24e−2) −	5.3427e−1 (1.34e−2) −	**5.0504e−1 (1.82e−2)**
		1000	5.4095e−1 (5.96e−4) −	5.3962e−1 (4.27e−3) −	**4.8963e−1 (4.16e−2)**
LSMOP6	2	500	6.0384e−1 (1.12e−1) −	**3.2041e−1 (8.17e−4) +**	4.2103e−1 (4.47e−2)
		1000	6.0757e−1 (1.15e−1) −	**3.1268e−1 (6.56e−4) +**	4.3718e−1 (8.52e−2)
	3	500	1.2872e+0 (4.33e−3) −	**7.2232e−1 (2.23e−2) +**	1.2853e+0 (1.12e−3)
		1000	1.3116e+0 (4.37e−3) −	**7.5947e−1 (4.11e−2) +**	1.3074e+0 (7.45e−4)
LSMOP7	2	500	1.5051e+0 (1.55e−3) −	**1.5011e+0 (1.39e−3) +**	1.5033e+0 (9.93e−4)
		1000	1.5145e+0 (1.50e−3) −	**1.5100e+0 (6.84e−4) +**	1.5110e+0 (5.87e−4)
	3	500	8.7506e−1 (1.20e−2) =	8.9463e−1 (9.93e−3) −	**8.7012e−1 (2.82e−3)**
		1000	8.5570e−1 (2.71e−3) −	8.6279e−1 (8.61e−3) −	**8.5039e−1 (9.58e−4)**
LSMOP8	2	500	6.4903e−1 (1.60e−1) −	7.4209e−1 (2.28e−16) −	**5.7144e−1 (1.05e−1)**
		1000	7.3533e−1 (2.09e−2) −	7.4209e−1 (2.28e−16) −	**6.6607e−1 (1.78e−1)**
	3	500	3.2674e−1 (3.85e−2) −	3.3954e−1 (2.77e−2) −	**2.3200e−1 (3.31e−2)**
		1000	3.2898e−1 (3.33e−2) −	3.5427e−1 (4.45e−2) −	**2.2963e−1 (2.93e−2)**
LSMOP9	2	500	8.0962e−1 (6.23e−4) −	8.0954e−1 (6.84e−4) −	**8.0848e−1 (1.03e−3)**
		1000	8.0827e−1 (1.62e−3) −	8.0799e−1 (1.59e−3) −	**8.0426e−1 (2.61e−3)**
	3	500	**1.1636e+0 (8.35e−2) =**	1.5183e+0 (8.79e−2) −	1.1647e+0 (8.78e−2)
		1000	**1.1447e+0 (4.01e−4) +**	1.4593e+0 (1.61e−1) −	1.1450e+0 (2.84e−4)
+/−/=			1/26/9	8/23/5	

#### Performance comparison of different optimization frameworks

To verify the efficacy of various large-scale multi-objective algorithm frameworks, NSGAII^28^ is incorporated into WOF^24^, LSMOF^18^ and MPSOF as the main optimizer. Subsequently, experiments are conducted on LSMOP, and the obtained results are compared for analysis.

Tables 5 and 6 display the average IGD and HV values achieved by NSGAII in the different algorithm frameworks.

**Table 6:** The statistical HV results with WOF-NSGAII, LS-NSGAII, and MPSNSGAII on 36 LSMOP test problems.

Problem	M	D	WOF-NSGAII	LS-NSGAII	MPSNSGAII
LSMOP1	2	500	9.7511e−2 (7.88e−3) −	1.0919e−1 (7.43e−3) −	**2.0263e−1 (9.02e−2)**
		1000	9.9717e−2 (1.66e−2) −	1.1079e−1 (6.76e−3) −	**2.1012e−1 (8.25e−2)**
	3	500	2.7921e−1 (6.02e−2) =	2.1019e−1 (9.89e−3) −	**3.1348e−1 (6.88e−2)**
		1000	1.6321e−1 (3.44e−2) −	1.4313e−1 (1.27e−2) −	**2.8984e−1 (8.50e−2)**
LSMOP2	2	500	5.4788e−1 (3.26e−3) −	5.5370e−1 (9.87e−4) =	**5.5430e−1 (2.94e−3)**
		1000	5.6037e−1 (4.50e−4) −	5.6121e−1 (6.34e−4) −	**5.6178e−1 (8.79e−4)**
	3	500	7.7174e−1 (4.06e−3) =	7.7183e−1 (3.62e−3) =	**7.7421e−1 (3.87e−3)**
		1000	7.9666e−1 (3.41e−3) =	7.9597e−1 (4.51e−3) =	**7.9680e−1 (3.60e−3)**
LSMOP3	2	500	0.0000e+0 (0.00e+0) =	0.0000e+0 (0.00e+0) =	0.0000e+0 (0.00e+0)
		1000	0.0000e+0 (0.00e+0) =	0.0000e+0 (0.00e+0) =	0.0000e+0 (0.00e+0)
	3	500	8.3649e−2 (2.08e−2) −	**9.0925e−2 (1.95e−5) =**	9.0913e−2 (6.84e−6)
		1000	8.8881e−2 (6.52e−3) −	**9.0923e−2 (1.69e−5) =**	9.0914e−2 (7.64e−6)
LSMOP4	2	500	4.9893e−1 (6.25e−3) −	**5.1581e−1 (1.34e−3) =**	5.1533e−1 (2.53e−3)
		1000	5.3082e−1 (3.96e−3) −	5.4088e−1 (1.43e−3) =	**5.4159e−1 (2.01e−3)**
	3	500	6.0792e−1 (7.23e−3) −	6.0199e−1 (9.02e−3) −	**6.1506e−1 (1.04e−2)**
		1000	7.0451e−1 (5.20e−3) =	7.0310e−1 (5.51e−3) −	**7.0717e−1 (4.98e−3)**
LSMOP5	2	500	9.0909e−2 (2.12e−7) −	9.0909e−2 (4.27e−17) =	**9.1991e−2 (4.84e−3)**
		1000	9.0905e−2 (1.03e−5) −	**9.0909e−2 (4.27e−17) =**	9.0909e−2 (4.27e−17)
	3	500	3.4450e−1 (2.22e−3) =	**3.4635e−1 (2.50e−4) +**	3.4458e−1 (5.52e−4)
		1000	3.4481e−1 (3.53e−4) =	**3.4646e−1 (2.63e−4) +**	3.4482e−1 (3.64e−4)
LSMOP6	2	500	5.3287e−2 (5.46e−3) −	3.5145e−2 (1.29e−3) −	**6.9013e−2 (2.74e−3)**
		1000	6.9573e−2 (3.61e−3) −	6.9593e−2 (1.48e−4) −	**8.0290e−2 (2.04e−3)**
	3	500	0.0000e+0 (0.00e+0) =	**1.2197e−2 (5.39e−3) +**	0.0000e+0 (0.00e+0)
		1000	0.0000e+0 (0.00e+0) =	**9.9542e−3 (6.96e−3) +**	0.0000e+0 (0.00e+0)
LSMOP7	2	500	0.0000e+0 (0.00e+0) =	0.0000e+0 (0.00e+0) =	0.0000e+0 (0.00e+0)
		1000	0.0000e+0 (0.00e+0) =	0.0000e+0 (0.00e+0) =	0.0000e+0 (0.00e+0)
	3	500	**2.3300e−4 (1.04e−3) =**	0.0000e+0 (0.00e+0) =	0.0000e+0 (0.00e+0)
		1000	3.7558e−3 (6.62e−3) −	0.0000e+0 (0.00e+0) −	**8.6613e−3 (3.51e−3)**
LSMOP8	2	500	9.1407e−2 (2.23e−3) −	9.0909e−2 (4.27e−17) =	**9.5849e−2 (2.19e−2)**
		1000	9.0909e−2 (5.02e−7) −	9.0909e−2 (4.27e−17) =	**1.0139e−1 (3.23e−2)**
	3	500	**3.7924e−1 (3.42e−2) +**	3.2018e−1 (7.97e−2) =	3.3941e−1 (3.21e−2)
		1000	**3.6352e−1 (6.03e−2) +**	2.6435e−1 (6.97e−2) −	3.4080e−1 (3.15e−2)
LSMOP9	2	500	9.0946e−2 (5.79e−5) −	9.0952e−2 (5.89e−5) −	**9.1102e−2 (1.30e−4)**
		1000	9.1110e−2 (2.09e−4) −	9.1144e−2 (1.83e−4) −	**9.1690e−2 (3.65e−4)**
	3	500	**1.4481e−1 (1.24e−2) +**	9.3745e−2 (1.27e−2) −	1.4477e−1 (1.27e−2)
		1000	**1.4760e−1 (1.32e−5) =**	1.0225e−1 (2.33e−2) −	1.4759e−1 (1.78e−5)
+/−/=			3/19/14	4/15/17	

Table 5 indicates that the three frameworks respectively yield the optimal values in 2, 9, and 25 out of 36 test instances. On the discontinuous LSMOP9 problem with 3-objective, WOF-NSGAII achieves the best results, but the differences between the three algorithms are not significant, indicating that none of the three algorithms has an absolute advantage in this class of problems. Additionally, MPSNSGAII performs slightly lower than LS-NSGAII on the LSMOP6 and LSMOP7 problems with 2-objective. From the last row of the test results, WOF-NSGAII and LS-NSGAII outperform MPSNSGAII on 1 and 8 test problems, are inferior to MPSNSGAII on 26 and 23 test problems, and show comparable performance to MPSNSGAII on 9 and 5 test problems, respectively. The comprehensive performance of MPSOF is the best. This is due to the introduction of an additional set of global weight updating populations, which effectively balances diversity and convergence.

Table 6 demonstrates that the overall performance of MPSNSGAII outperforms the other two comparative frameworks. Specifically, WOF-NSGAII, LS-NSGAII and MPSNSGAII achieve 5, 8, and 19 best results respectively. Among them, LS-NSGAII performed well on the 3-objective LSMOP3 and LSMOP5 problems, However, the similar HV values between MPSNSGAII and LS-NSGAII indicate comparable performance in handling these two types of problems. For the 2-objective LSMOP3 problem, all three algorithms result in HV values of zero, indicating that none of the compared algorithms demonstrate ideal performance. For 36 test instances, WOF-NSGAII outperforms MPSNSGAII on 3 instances, is inferior to MPSNSGAII on 19 instances, and shows similar performance to the algorithms in this paper on 14 instances. LS-NSGAII outperforms MPSNSGAII on 4 instances, is inferior to MPSNSGAII on 15 instances, and shows similar performance to MPSNSGAII on 17 instances.

Regardless of the IGD or HV values, MPSNSGAII achieves optimal results in most instances, indicating a clear advantage of the proposed framework over the other two algorithm frameworks.

#### Performance comparison of cutting-edge large-scale MOEAs

To compare the performance of MPSOF with several advanced algorithms, LMOCSO is integrated into MPSOF and named MPSLMOCSO. Subsequently, MPSLMOCSO is compared with seven other algorithms (LSTPA^34^, DGEA^35^, SSCEA^36^, IM-MOEA/D^21^, FDV^37^, ATLMOEA^38^ and LERD^39^) on three function sets of LSMOP, WFG, and UF.

These algorithms represent the three types of methods currently used to solve LSMOPs. Specifically, LSTPA is a CSO that accelerates convergence using fitness values. DGEA is a direction-guided adaptive reproduction algorithm. SSCEA is a CC algorithm that performs segmentation processing on subspaces. IM-MOEA/D is a distribution estimation algorithm that can effectively solve LSMOPs. FDV belongs to the problem transformation algorithms, which processes decision variables through fuzzy and precise evolution. ATLMOEA is an adaptive two-stage algorithm that uses neural networks to accelerate optimization. LERD is an improved algorithm for DVA that greatly reduces the cost of function evaluation required for DVA.

**Figure 4:**
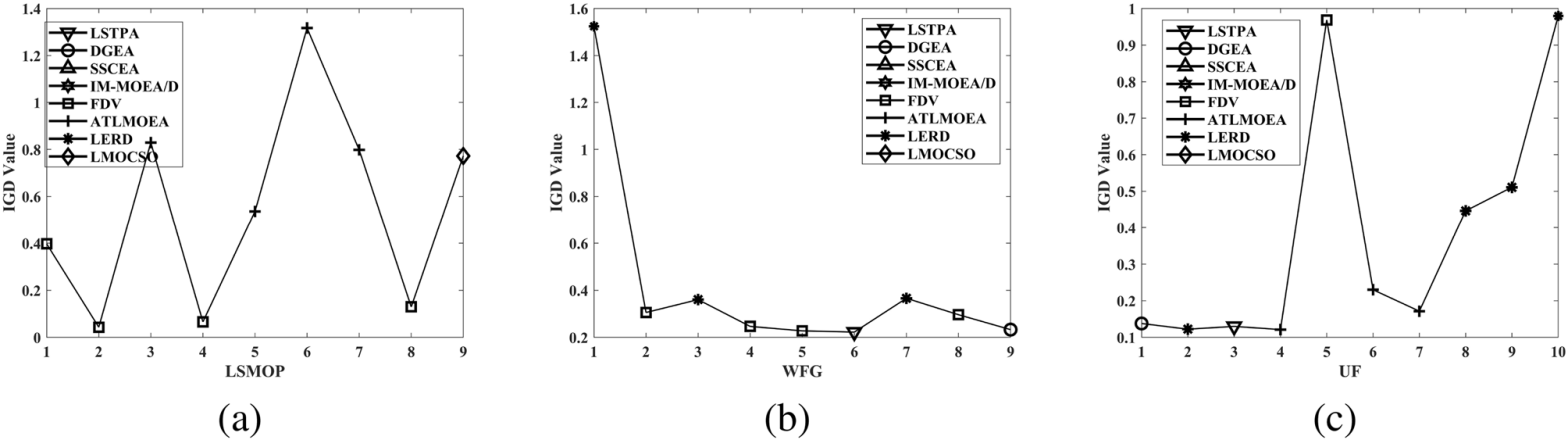
The performance statistics of eight comparison algorithms with three function sets.

To validate the efficacy of MPSLMOCSO, LMOCSO is first compared with other seven algorithms. The statistical results of the algorithms that achieve optimal average IGD values on LSMOP1-LSMOP9, WFG1-WFG9, and UF1-UF10 are presented in Fig. 4.

**Table 7:** The statistical IGD results with LSTPA, DGEA, SSCEA, IM-MOEA/D, FDV ,ATLMOEA, LERD and MPSLMOCSO on 36 LSMOP test problems.

Problem	M	D	LSTPA	DGEA	SSCEA	IM-MOEA/D	FDV	ATLMOEA	LERD	MPSLMOCSO
LSMOP1	2	500	2.4414e−1 (6.90e−2) −	3.3734e−1 (5.36e−2) −	1.0821e+1 (4.17e−1) −	5.5801e−1 (2.50e−2) −	1.5834e−1 (1.98e−2) −	3.3542e−1 (4.64e−2) −	9.4674e−1 (1.49e−1) −	**8.2846e−2 (4.79e−2)**
		1000	3.8949e−1 (1.52e−1) −	3.5353e−1 (4.30e−2) −	1.1117e+1 (1.65e−1) −	1.9237e+0 (3.13e−1) −	2.7658e−1 (1.32e−2) −	3.7388e−1 (5.77e−2) −	1.3869e+0 (1.42e−1) −	**8.1982e−2 (3.19e−2)**
	3	500	5.5832e−1 (4.75e−2) −	6.3932e−1 (1.35e−1) −	1.1080e+1 (5.05e−1) −	1.1858e+0 (4.73e−1) −	3.4478e−1 (2.95e−2) −	5.7492e−1 (8.19e−2) −	1.1777e+0 (2.93e−1) −	**1.5729e−1 (4.84e−2)**
		1000	6.1347e−1 (8.89e−2) −	6.1478e−1 (1.20e−1) −	1.1386e+1 (3.51e−1) −	4.5828e+0 (6.19e−1) −	3.9748e−1 (2.68e−2) −	6.8138e−1 (8.91e−2) −	1.4556e+0 (1.44e−1) −	**1.3813e−1 (2.67e−2)**
LSMOP2	2	500	1.7084e−2 (1.18e−2) −	1.4201e−2 (6.65e−4) −	7.3869e−2 (3.02e−4) −	5.4312e−2 (6.82e−4) −	1.9692e−2 (5.87e−3) −	4.0586e−2 (1.66e−3) −	3.6254e−2 (5.11e−3) −	**5.6351e−3 (2.48e−4)**
		1000	1.9864e−2 (7.98e−3) −	8.6516e−3 (6.50e−4) −	4.0737e−2 (3.72e−4) −	3.6029e−2 (2.86e−4) −	1.5509e−2 (3.96e−3) −	2.3333e−2 (8.57e−4) −	2.2441e−2 (1.08e−3) −	**3.5193e−3 (1.29e−4)**
	3	500	5.9934e−2 (7.68e−4) −	5.3955e−2 (2.10e−3) −	7.9308e−2 (2.36e−3) −	7.3562e−2 (6.65e−4) −	**4.9658e−2 (1.87e−3) +**	5.6769e−2 (8.05e−4) −	6.7296e−2 (1.19e−3) −	5.1420e−2 (1.57e−3)
		1000	4.9341e−2 (4.35e−4) −	4.5655e−2 (9.73e−4) −	6.3102e−2 (1.80e−3) −	6.1283e−2 (1.05e−3) −	4.3112e−2 (4.56e−4) −	4.7286e−2 (1.45e−3) −	5.0931e−2 (2.66e−4) −	**2.6768e−2 (5.11e−4)**
LSMOP3	2	500	4.1096e+0 (3.01e+0) −	1.3358e+0 (2.98e−1) =	1.6457e+3 (1.16e+3) −	3.1598e+0 (6.48e−1) −	**1.0100e+0 (4.82e−2) +**	1.5696e+0 (5.51e−3) −	1.1660e+1 (4.86e+0) −	1.2556e+0 (1.10e−1)
		1000	4.9163e+0 (2.66e+0) −	1.7516e+0 (8.41e−1) −	1.1453e+3 (1.35e+3) −	1.1262e+1 (2.70e+0) −	1.4222e+0 (7.27e−2) −	1.5802e+0 (3.23e−3) −	1.3860e+1 (2.66e+0) −	**1.1831e+0 (2.94e−1)**
	3	500	1.6272e+0 (1.38e+0) −	9.3296e−1 (5.41e−1) =	4.9235e+2 (5.16e+2) −	6.4262e+0 (1.38e+0) −	1.3589e+0 (7.95e−1) −	8.2573e−1 (3.64e−2) =	9.5808e+0 (4.51e−1) −	**8.2089e−1 (2.08e−2)**
		1000	2.4685e+0 (2.34e+0) −	9.0869e−1 (2.22e−1) =	2.7418e+2 (2.70e+2) −	1.1272e+1 (2.05e+0) −	1.6522e+0 (8.12e−1) −	**8.2891e−1 (3.68e−2) =**	1.0019e+1 (4.92e−1) −	8.5945e−1 (3.46e−3)
LSMOP4	2	500	3.5772e−2 (2.28e−3) −	4.6757e−2 (2.67e−3) −	1.3653e−1 (5.10e−4) −	6.2922e−2 (2.07e−3) −	3.7842e−2 (2.98e−3) −	5.2622e−2 (6.50e−3) −	5.9453e−2 (5.04e−3) −	**3.2578e−2 (6.41e−4)**
		1000	2.4910e−2 (6.57e−3) −	2.5407e−2 (1.54e−3) −	7.8202e−2 (3.36e−4) −	5.0316e−2 (1.10e−3) −	2.2556e−2 (1.07e−3) −	3.5461e−2 (4.64e−3) −	3.6608e−2 (3.52e−3) −	**1.7460e−2 (3.01e−4)**
	3	500	1.5565e−1 (1.98e−3) −	1.1859e−1 (4.06e−3) −	2.1485e−1 (3.40e−3) −	1.6545e−1 (2.58e−3) −	1.0411e−1 (5.59e−3) −	1.3064e−1 (6.46e−3) −	1.5699e−1 (6.02e−3) −	**9.0645e−2 (3.59e−3)**
		1000	9.8718e−2 (1.13e−3) −	7.4430e−2 (1.91e−3) −	1.3340e−1 (4.80e−3) −	1.1158e−1 (1.31e−3) −	6.6016e−2 (2.38e−3) −	8.7555e−2 (4.25e−3) −	1.0493e−1 (3.08e−3) −	**5.2447e−2 (1.33e−3)**
LSMOP5	2	500	1.5508e+0 (3.09e−1) −	2.2539e+0 (1.33e+0) −	2.3383e+1 (7.27e−1) −	8.5264e−1 (8.82e−2) −	**2.3272e−1 (1.93e−1) =**	7.4209e−1 (2.28e−16) −	2.6034e+0 (9.49e−1) −	2.3576e−1 (6.89e−2)
		1000	2.7170e+0 (4.97e−1) −	3.2631e+0 (1.61e+0) −	2.3840e+1 (4.46e−1) −	2.8388e+0 (3.96e−1) −	9.7187e−1 (3.90e−1) −	7.4209e−1 (2.28e−16) −	4.4385e+0 (1.24e+0) −	**2.7719e−1 (1.22e−1)**
	3	500	7.5821e−1 (3.43e−1) −	5.6358e−1 (2.17e−1) −	1.9270e+1 (9.58e−1) −	8.6070e−1 (7.84e−2) −	4.2868e−1 (2.22e−1) =	5.2727e−1 (1.77e−2) −	1.7845e+0 (1.27e+0) −	**3.0377e−1 (6.78e−2)**
		1000	1.0437e+0 (6.73e−1) −	6.1351e−1 (2.13e−1) −	1.9836e+1 (7.48e−1) −	2.0143e+0 (4.57e−1) −	5.4426e−1 (2.36e−1) −	5.3657e−1 (1.07e−2) −	2.9700e+0 (8.16e−1) −	**2.9252e−1 (1.26e−1)**
LSMOP6	2	500	7.8325e−1 (6.46e−5) −	5.6727e−1 (2.29e−1) −	1.8820e+3 (2.00e+3) −	6.9480e−1 (9.49e−2) −	7.7610e−1 (1.23e−3) −	6.1034e−1 (1.21e−1) −	7.5026e−1 (4.92e−2) −	**3.8639e−1 (1.59e−1)**
		1000	7.6066e−1 (7.48e−6) −	6.4655e−1 (1.83e−1) −	3.4737e+3 (3.16e+3) −	6.4253e−1 (1.11e−1) −	7.5975e−1 (1.56e−4) −	5.5607e−1 (1.52e−1) −	7.2915e−1 (5.82e−2) −	**3.5678e−1 (1.20e−1)**
	3	500	9.6950e+1 (7.70e+1) −	9.4620e+0 (2.07e+1) =	3.6090e+4 (4.52e+3) −	1.9366e+1 (6.26e+0) −	1.2166e+0 (6.76e−1) =	1.2964e+0 (7.13e−4) −	8.0606e+0 (9.47e+0) −	**9.4839e−1 (2.55e−1)**
		1000	2.0952e+2 (2.74e+2) −	2.6290e+1 (3.73e+1) =	3.8917e+4 (5.09e+3) −	1.9306e+2 (9.20e+1) −	1.4287e+0 (6.62e−1) −	1.3169e+0 (7.44e−4) −	9.7508e+1 (1.75e+2) −	**8.2596e−1 (2.16e−1)**
LSMOP7	2	500	5.6508e+2 (4.72e+2) −	1.2909e+3 (1.04e+3) −	8.1128e+4 (5.12e+3) −	9.3698e+1 (6.51e+1) −	2.6027e+0 (3.49e+0) −	1.5065e+0 (3.74e−3) −	4.0955e+0 (6.15e−1) −	**1.4829e+0 (6.40e−2)**
		1000	3.8523e+3 (1.10e+4) −	1.8085e+3 (1.61e+3) −	8.6727e+4 (4.56e+3) −	1.8952e+3 (1.45e+3) −	7.9752e+0 (2.80e+1) −	1.5138e+0 (2.53e−3) −	6.8837e+0 (1.51e+0) −	**1.4780e+0 (7.02e−2)**
	3	500	9.6015e−1 (5.99e−2) −	9.2463e−1 (6.57e−2) −	2.5892e+3 (2.38e+3) −	8.9941e−1 (1.10e−1) −	9.4986e−1 (1.00e−2) −	8.4443e−1 (3.68e−2) =	1.0264e+0 (1.18e−1) −	**8.4199e−1 (5.84e−2)**
		1000	9.2592e−1 (7.64e−2) −	9.2063e−1 (6.71e−2) −	2.1502e+3 (2.30e+3) −	8.6337e−1 (9.54e−2) =	9.6016e−1 (1.55e−2) −	7.9786e−1 (6.21e−2) =	1.0210e+0 (3.96e−2) −	**7.9596e−1 (1.02e−1)**
LSMOP8	2	500	1.5203e+0 (5.91e−1) −	8.4755e−1 (1.87e−1) −	1.9519e+1 (4.84e−1) −	3.4617e−1 (5.57e−2) −	**6.7083e−2 (2.06e−2) +**	7.3649e−1 (2.51e−2) −	6.4476e−1 (2.09e−1) −	1.3635e−1 (4.62e−2)
		1000	2.4008e+0 (5.61e−1) −	1.4056e+0 (8.68e−1) −	2.0201e+1 (3.48e−1) −	1.4154e+0 (1.76e−1) −	2.4687e−1 (5.19e−2) −	7.4209e−1 (2.28e−16) −	1.6526e+0 (3.90e−1) −	**1.6998e−1 (1.28e−1)**
	3	500	2.6618e−1 (5.92e−2) −	2.4242e−1 (1.68e−1) −	7.6337e−1 (1.13e−1) −	7.1800e−1 (9.47e−2) −	1.0752e−1 (6.28e−2) −	2.6457e−1 (2.16e−2) −	2.9795e−1 (4.97e−2) −	**7.3578e−2 (9.04e−3)**
		1000	2.2916e−1 (1.44e−2) −	2.1263e−1 (1.83e−1) −	8.3792e−1 (3.62e−1) −	6.1882e−1 (6.14e−2) −	1.2966e−1 (9.87e−2) −	2.7816e−1 (2.34e−2) −	3.6467e−1 (1.94e−1) −	**4.6251e−2 (1.03e−2)**
LSMOP9	2	500	6.4629e−1 (7.16e−2) −	1.9357e+0 (9.51e−1) −	5.6159e+1 (3.13e+0) −	1.1367e+0 (2.13e−1) −	**1.2737e−1 (2.18e−2) +**	8.4595e−1 (3.04e−2) −	1.1579e+0 (7.66e−1) −	1.4863e−1 (1.81e−1)
		1000	2.7616e+0 (7.52e−1) −	6.7866e+0 (2.21e+0) −	5.9899e+1 (1.46e+0) −	1.7342e+1 (1.42e+0) −	**1.4165e−1 (9.04e−2) +**	8.4755e−1 (2.32e−2) −	1.2367e+0 (8.19e−1) −	1.6827e−1 (2.10e−1)
	3	500	5.3374e+0 (6.94e−1) −	1.1569e+1 (3.14e+0) −	1.3778e+2 (7.02e+0) −	1.6799e+0 (4.57e−1) −	**5.2422e−1 (2.98e−2) +**	1.1946e+0 (4.40e−2) −	1.7363e+0 (3.49e−1) −	1.1470e+0 (1.35e−3)
		1000	1.8146e+1 (5.57e+0) −	2.4636e+1 (1.02e+1) −	1.4335e+2 (4.33e+0) −	1.0640e+1 (1.96e+0) −	1.9702e+0 (5.45e−1) −	1.2044e+0 (1.55e−1) −	2.9562e+0 (1.10e+0) −	**1.1441e+0 (4.83e−3)**
+/−/=			0/36/0	0/31/5	0/36/0	0/35/1	6/27/3	0/32/4	0/36/0

**Table 8:** The statistical HV results with LATPA, DGEA, SSCEA, IM-OEA/D, FDV, ATLMOEA, LERD and MPSLMOCSO on 36 LSMOP test problems.

problem	M	D	LSTPA	DGEA	SSCEA	IM-MOEA/D	FDV	ATLMOEA	LERD	MPSLMOCSO
LSMOP1	2	500	2.2154e−1 (4.75e−2) −	2.1589e−1 (4.42e−2) −	0.0000e+0 (0.00e+0) −	4.5236e−2 (1.77e−2) −	3.9367e−1 (2.15e−2) −	2.0133e−1 (3.00e−2) −	2.6868e−3 (7.01e−3) −	**4.8366e−1 (5.52e−2)**
		1000	1.4277e−1 (7.94e−2) −	2.1599e−1 (3.41e−2) −	0.0000e+0 (0.00e+0) −	0.0000e+0 (0.00e+0) −	2.8768e−1 (1.36e−2) −	2.0915e−1 (2.99e−2) −	0.0000e+0 (0.00e+0) −	**4.8590e−1 (3.50e−2)**
	3	500	1.6559e−1 (4.94e−2) −	1.3873e−1 (9.99e−2) −	0.0000e+0 (0.00e+0) −	1.0940e−2 (2.39e−2) −	4.1420e−1 (3.12e−2) −	1.7040e−1 (6.37e−2) −	2.5436e−2 (5.30e−2) −	**7.1462e−1 (2.20e−2)**
		1000	1.3975e−1 (7.04e−2) −	1.6855e−1 (9.47e−2) −	0.0000e+0 (0.00e+0) −	0.0000e+0 (0.00e+0) −	3.6175e−1 (2.98e−2) −	1.3630e−1 (4.40e−2) −	0.0000e+0 (0.00e+0) −	**6.9746e−1 (4.52e−2)**
LSMOP2	2	500	5.6284e−1 (1.56e−2) −	5.6686e−1 (8.39e−4) −	4.8668e−1 (1.23e−3) −	5.1341e−1 (8.45e−4) −	5.5967e−1 (7.68e−3) −	5.3182e−1 (2.21e−3) −	5.3777e−1 (6.90e−3) −	**5.7934e−1 (3.08e−4)**
		1000	5.5887e−1 (1.07e−2) −	5.7386e−1 (8.55e−4) −	5.2874e−1 (1.25e−3) −	5.3734e−1 (5.81e−4) −	5.6470e−1 (5.20e−3) −	5.5441e−1 (1.16e−3) −	5.5578e−1 (1.39e−3) −	**5.8194e−1 (1.56e−4)**
	3	500	8.0503e−1 (2.06e−3) −	8.1605e−1 (2.78e−3) −	7.6342e−1 (5.53e−3) −	7.5332e−1 (4.94e−3) −	8.2206e−1 (2.64e−3) −	8.1101e−1 (1.24e−3) −	7.9921e−1 (1.52e−3) −	**8.3751e−1 (1.52e−3)**
		1000	8.2141e−1 (9.47e−4) −	8.2741e−1 (1.60e−3) −	7.8654e−1 (4.66e−3) −	7.7695e−1 (4.71e−3) −	8.3151e−1 (7.87e−4) −	8.2478e−1 (1.55e−3) −	8.2007e−1 (3.24e−4) −	**8.4744e−1 (5.49e−4)**
LSMOP3	2	500	0.0000e+0 (0.00e+0) =	0.0000e+0 (0.00e+0) =	0.0000e+0 (0.00e+0) =	0.0000e+0 (0.00e+0) =	0.0000e+0 (0.00e+0) =	0.0000e+0 (0.00e+0) =	0.0000e+0 (0.00e+0) =	0.0000e+0 (0.00e+0)
		1000	0.0000e+0 (0.00e+0) =	0.0000e+0 (0.00e+0) =	0.0000e+0 (0.00e+0) =	0.0000e+0 (0.00e+0) =	0.0000e+0 (0.00e+0) =	0.0000e+0 (0.00e+0) =	0.0000e+0 (0.00e+0) =	0.0000e+0 (0.00e+0)
	3	500	6.8182e−2 (4.04e−2) −	8.6385e−2 (2.03e−2) −	0.0000e+0 (0.00e+0) −	0.0000e+0 (0.00e+0) −	5.3240e−2 (4.50e−2) −	**9.0913e−2 (5.45e−6) =**	0.0000e+0 (0.00e+0) −	9.0910e−2 (3.37e−6)
		1000	5.4546e−2 (4.57e−2) −	8.1821e−2 (2.80e−2) =	0.0000e+0 (0.00e+0) −	0.0000e+0 (0.00e+0) −	4.0909e−2 (4.64e−2) −	**9.0932e−2 (8.07e−5) +**	0.0000e+0 (0.00e+0) −	9.0909e−2 (1.84e−7)
LSMOP4	2	500	5.3830e−1 (2.92e−3) −	5.2433e−1 (3.31e−3) −	4.1475e−1 (1.01e−3) −	5.0024e−1 (2.41e−3) −	5.3581e−1 (3.72e−3) −	5.1679e−1 (8.06e−3) −	5.0845e−1 (6.22e−3) −	**5.4484e−1 (7.66e−4)**
		1000	5.5260e−1 (8.67e−3) −	5.5205e−1 (2.10e−3) −	4.8159e−1 (9.65e−4) −	5.1733e−1 (1.40e−3) −	5.5588e−1 (1.34e−3) −	5.3827e−1 (6.30e−3) −	5.3674e−1 (4.65e−3) −	**5.6474e−1 (3.71e−4)**
	3	500	6.7991e−1 (2.56e−3) −	7.4374e−1 (4.91e−3) −	5.8112e−1 (8.12e−3) −	6.4129e−1 (4.75e−3) −	7.6012e−1 (7.01e−3) −	7.2239e−1 (9.45e−3) −	6.8975e−1 (8.89e−3) −	**7.9382e−1 (2.18e−3)**
		1000	7.5369e−1 (2.24e−3) −	7.9259e−1 (2.56e−3) −	6.8744e−1 (8.39e−3) −	7.0895e−1 (5.41e−3) −	8.0295e−1 (3.27e−3) −	7.7162e−1 (6.03e−3) −	7.5411e−1 (3.69e−3) −	**8.2206e−1 (1.33e−3)**
LSMOP5	2	500	9.0909e−3 (2.80e−2) −	1.7671e−2 (3.63e−2) −	0.0000e+0 (0.00e+0) −	0.0000e+0 (0.00e+0) −	**1.4480e−1 (7.22e−2) =**	9.0909e−2 (4.27e−17) −	4.5455e−3 (2.03e−2) −	1.4353e−1 (4.50e−2)
		1000	4.5455e−3 (2.03e−2) −	9.0909e−3 (2.80e−2) −	0.0000e+0 (0.00e+0) −	0.0000e+0 (0.00e+0) −	1.7826e−2 (3.66e−2) −	9.0909e−2 (4.27e−17) −	0.0000e+0 (0.00e+0) −	**1.3981e−1 (5.42e−2)**
	3	500	1.8089e−1 (1.60e−1) −	3.0781e−1 (7.25e−2) −	0.0000e+0 (0.00e+0) −	6.5199e−3 (9.52e−3) −	2.7998e−1 (1.09e−1) −	3.2330e−1 (3.88e−4) −	8.4917e−2 (1.21e−1) −	**3.6789e−1 (3.77e−2)**
		1000	1.6057e−1 (1.53e−1) −	2.8003e−1 (1.09e−1) −	0.0000e+0 (0.00e+0) −	0.0000e+0 (0.00e+0) −	2.5525e−1 (1.24e−1) −	3.2290e−1 (9.69e−4) −	0.0000e+0 (0.00e+0) −	**3.7310e−1 (4.11e−2)**
LSMOP6	2	500	1.4508e−2 (1.03e−4) −	3.8965e−2 (2.23e−2) −	1.5362e−2 (8.28e−3) −	0.0000e+0 (0.00e+0) −	2.6142e−2 (2.04e−3) −	2.5405e−2 (9.11e−3) −	9.4967e−3 (8.10e−3) −	**9.3337e−2 (2.03e−2)**
		1000	5.2924e−2 (1.37e−5) −	6.0420e−2 (8.29e−3) −	0.0000e+0 (0.00e+0) −	3.1097e−2 (8.70e−4) −	5.4579e−2 (2.88e−4) −	5.5130e−2 (1.73e−3) −	4.4376e−2 (4.44e−3) −	**1.0453e−1 (1.57e−2)**
	3	500	0.0000e+0 (0.00e+0) −	1.3646e−2 (1.68e−2) =	0.0000e+0 (0.00e+0) −	0.0000e+0 (0.00e+0) −	1.7778e−2 (2.25e−2) =	0.0000e+0 (0.00e+0) −	0.0000e+0 (0.00e+0) −	**3.3925e−2 (4.61e−2)**
		1000	0.0000e+0 (0.00e+0) −	1.6750e−2 (2.12e−2) −	0.0000e+0 (0.00e+0) −	0.0000e+0 (0.00e+0) −	6.9082e−3 (1.09e−2) −	0.0000e+0 (0.00e+0) −	0.0000e+0 (0.00e+0) −	**7.0928e−2 (5.49e−2)**
LSMOP7	2	500	0.0000e+0 (0.00e+0) =	0.0000e+0 (0.00e+0) =	0.0000e+0 (0.00e+0) =	0.0000e+0 (0.00e+0) =	0.0000e+0 (0.00e+0) =	0.0000e+0 (0.00e+0) =	0.0000e+0 (0.00e+0) =	0.0000e+0 (0.00e+0)
		1000	0.0000e+0 (0.00e+0) =	0.0000e+0 (0.00e+0) =	0.0000e+0 (0.00e+0) =	0.0000e+0 (0.00e+0) =	0.0000e+0 (0.00e+0) =	0.0000e+0 (0.00e+0) =	0.0000e+0 (0.00e+0) =	0.0000e+0 (0.00e+0)
	3	500	3.7674e−3 (1.54e−2) −	9.4595e−5 (4.23e−4) −	0.0000e+0 (0.00e+0) −	3.7820e−3 (5.30e−3) =	**8.3835e−2 (1.72e−2) +**	0.0000e+0 (0.00e+0) −	1.5762e−3 (7.05e−3) −	1.1097e−2 (2.01e−2)
		1000	1.5735e−2 (1.01e−2) −	1.0219e−2 (9.07e−3) −	0.0000e+0 (0.00e+0) −	1.2056e−4 (4.24e−4) −	**6.5549e−2 (2.69e−2) +**	1.7496e−3 (2.83e−3) −	1.6626e−4 (7.44e−4) −	2.6521e−2 (9.42e−3)
LSMOP8	2	500	1.3568e−2 (3.23e−2) −	4.5989e−2 (4.03e−2) −	0.0000e+0 (0.00e+0) −	8.2285e−2 (1.80e−2) −	**2.6401e−1 (1.92e−2) +**	9.0909e−2 (4.27e−17) −	2.9318e−2 (3.78e−2) −	2.1990e−1 (2.57e−2)
		1000	4.4655e−3 (2.00e−2) −	3.3956e−2 (4.29e−2) −	0.0000e+0 (0.00e+0) −	0.0000e+0 (0.00e+0) −	1.5197e−1 (3.07e−2) −	9.0909e−2 (4.27e−17) −	0.0000e+0 (0.00e+0) −	**2.1246e−1 (3.26e−2)**
	3	500	3.5993e−1 (4.83e−2) −	3.5187e−1 (1.19e−1) −	3.3497e−2 (1.28e−2) −	8.0324e−2 (3.65e−3) −	4.4698e−1 (6.21e−2) −	3.6935e−1 (4.73e−3) −	3.5614e−1 (1.23e−2) −	**5.3277e−1 (1.84e−2)**
		1000	3.7876e−1 (3.72e−3) −	3.8255e−1 (9.03e−2) −	3.4316e−2 (2.54e−2) −	7.7181e−2 (9.76e−4) −	4.2364e−1 (9.78e−2) −	3.7561e−1 (2.26e−3) −	2.6563e−1 (1.43e−1) −	**5.3521e−1 (1.81e−2)**
LSMOP9	2	500	4.4664e−2 (6.47e−3) −	2.0655e−3 (4.23e−3) −	0.0000e+0 (0.00e+0) −	5.3356e−3 (5.89e−3) −	1.8349e−1 (9.34e−3) −	8.0868e−2 (8.23e−3) −	5.9749e−2 (3.24e−2) −	**2.0297e−1 (2.39e−2)**
		1000	0.0000e+0 (0.00e+0) −	0.0000e+0 (0.00e+0) −	0.0000e+0 (0.00e+0) −	0.0000e+0 (0.00e+0) −	1.8084e−1 (3.39e−2) −	8.0207e−2 (6.42e−3) −	3.5396e−2 (2.09e−2) −	**2.0697e−1 (2.95e−2)**
	3	500	0.0000e+0 (0.00e+0) −	0.0000e+0 (0.00e+0) −	0.0000e+0 (0.00e+0) −	3.7913e−3 (4.12e−3) −	5.1760e−2 (7.05e−3) −	1.3674e−1 (9.07e−3) −	5.0985e−2 (3.66e−2) −	**1.4736e−1 (1.56e−4)**
		1000	0.0000e+0 (0.00e+0) −	0.0000e+0 (0.00e+0) −	0.0000e+0 (0.00e+0) −	0.0000e+0 (0.00e+0) −	3.0353e−3 (5.62e−3) −	1.3140e−1 (1.72e−2) −	4.8129e−3 (1.49e−2) −	**1.4742e−1 (6.02e−4)**
+/−/=			0/32/4	0/30/6	0/32/4	0/31/5	3/27/6	1/30/5	0/32/4

**Table 9:** The statistical IGD results with LSTPA, DGEA, SSCEA, IM-MOEA/D, FDV, ATLMOEA, LERD and MPSLMOCSO on 36 WFG test problems.

Problem	M	D	LSTPA	DGEA	SSCEA	IM-MOEA/D	FDV	ATLMOEA	LERD	MPSLMOCSO
WFG1	2	500	1.3422e+0 (2.27e−2) −	1.3542e+0 (9.76e−2) −	2.0259e+0 (9.06e−2) −	1.6061e+0 (2.05e−1) −	**1.1937e+0 (2.43e−1) +**	1.3343e+0 (2.61e−2) −	1.2554e+0 (6.49e−3) +	1.2430e+0 (1.20e−2)
		1000	1.3620e+0 (2.05e−2) −	1.3549e+0 (8.11e−2) −	2.0455e+0 (8.44e−2) −	1.7731e+0 (1.52e−1) −	1.3689e+0 (1.80e−1) =	1.3372e+0 (2.39e−2) −	1.2698e+0 (4.85e−3) −	**1.2669e+0 (5.80e−2)**
	3	500	1.5698e+0 (2.70e−2) −	1.5427e+0 (1.71e−2) −	2.3977e+0 (5.92e−2) −	1.7265e+0 (6.38e−2) −	1.5057e+0 (1.85e−1) =	1.5617e+0 (2.46e−2) −	1.5279e+0 (1.27e−2) −	**1.4082e+0 (1.60e−1)**
		1000	1.5897e+0 (1.97e−2) −	1.5421e+0 (1.30e−2) −	2.4040e+0 (7.33e−2) −	1.9202e+0 (8.33e−2) −	1.6108e+0 (1.31e−1) −	1.5613e+0 (2.18e−2) −	1.5244e+0 (1.42e−2) −	**1.4419e+0 (6.65e−2)**
WFG2	2	500	2.2753e−1 (1.56e−2) −	2.9337e−1 (2.43e−2) −	6.8357e−1 (2.64e−2) −	2.0851e−1 (1.17e−2) −	1.8459e−1 (9.69e−3) −	2.5200e−1 (1.93e−2) −	1.8375e−1 (2.00e−2) −	**1.0665e−1 (8.93e−3)**
		1000	2.5545e−1 (2.23e−2) −	3.0633e−1 (1.20e−2) −	7.0019e−1 (3.37e−2) −	2.9672e−1 (1.51e−2) −	1.8244e−1 (7.83e−3) −	2.7565e−1 (1.52e−2) −	2.2799e−1 (1.54e−2) −	**1.1457e−1 (8.00e−3)**
	3	500	3.5557e−1 (3.08e−2) −	4.4603e−1 (2.55e−2) −	1.0818e+0 (1.37e−1) −	4.3982e−1 (1.51e−2) −	2.7498e−1 (6.16e−3) −	3.6446e−1 (2.63e−2) −	3.4928e−1 (1.47e−2) −	**2.4617e−1 (2.28e−2)**
		1000	3.8046e−1 (2.63e−2) −	4.4257e−1 (2.04e−2) −	1.0711e+0 (1.11e−1) −	5.0122e−1 (1.75e−2) −	3.0560e−1 (7.37e−3) −	3.8142e−1 (2.19e−2) −	3.7852e−1 (1.99e−2) −	**2.5242e−1 (2.42e−2)**
WFG3	2	500	2.4074e−1 (6.44e−3) −	3.4672e−1 (1.16e−2) −	7.2669e−1 (2.25e−3) −	2.1957e−1 (1.46e−2) −	1.8872e−1 (8.22e−3) −	2.6195e−1 (9.50e−3) −	2.5172e−1 (1.86e−2) −	**9.1852e−2 (9.08e−3)**
		1000	2.7541e−1 (7.25e−3) −	3.7434e−1 (1.69e−2) −	7.3513e−1 (1.85e−3) −	3.0642e−1 (1.38e−2) −	1.9125e−1 (4.79e−3) −	2.8100e−1 (6.56e−3) −	2.8440e−1 (7.65e−3) −	**9.3146e−2 (9.47e−3)**
	3	500	4.3308e−1 (2.84e−2) −	4.5546e−1 (2.54e−2) −	8.8700e−1 (6.70e−3) −	4.6493e−1 (1.72e−2) −	3.9834e−1 (1.55e−2) −	4.3824e−1 (2.07e−2) −	3.4237e−1 (1.90e−2) −	**2.8412e−1 (3.40e−2)**
		1000	4.6183e−1 (3.06e−2) −	4.6643e−1 (3.92e−2) −	8.9196e−1 (5.58e−3) −	5.8285e−1 (2.30e−2) −	4.0950e−1 (1.83e−2) −	4.5081e−1 (2.37e−2) −	3.6039e−1 (2.09e−2) −	**2.8356e−1 (4.36e−2)**
WFG4	2	500	1.4077e−1 (3.44e−3) −	1.9314e−1 (1.39e−2) −	4.7358e−1 (2.71e−2) −	8.4299e−2 (4.25e−3) +	**6.2791e−2 (8.49e−4) +**	1.6958e−1 (4.23e−3) −	1.5354e−1 (1.58e−2) −	1.0759e−1 (3.04e−3)
		1000	1.5460e−1 (3.42e−3) −	2.0263e−1 (1.17e−2) −	4.6411e−1 (1.21e−2) −	1.3483e−1 (7.30e−3) −	**7.2248e−2 (2.20e−3) +**	1.7132e−1 (3.03e−3) −	1.7479e−1 (1.09e−2) −	1.1163e−1 (3.04e−3)
	3	500	2.9971e−1 (4.39e−3) −	3.2866e−1 (1.01e−2) −	8.4202e−1 (8.23e−2) −	3.6756e−1 (8.92e−3) −	**2.4019e−1 (2.58e−3) +**	3.0087e−1 (6.95e−3) −	3.2706e−1 (8.33e−3) −	2.5599e−1 (3.64e−2)
		1000	3.0496e−1 (6.77e−3) −	3.2734e−1 (1.26e−2) −	8.5479e−1 (7.31e−2) −	4.0870e−1 (1.10e−2) −	**2.4658e−1 (3.07e−3) +**	3.0717e−1 (9.04e−3) −	3.2229e−1 (9.29e−3) −	2.5670e−1 (3.56e−2)
WFG5	2	500	6.5195e−2 (1.38e−3) =	6.3907e−2 (3.40e−4) +	6.6092e−1 (5.56e−3) −	2.8752e−1 (2.12e−2) −	**6.3900e−2 (3.20e−4) +**	7.2213e−2 (4.31e−3) −	6.7118e−2 (2.09e−3) −	6.4080e−2 (2.81e−3)
		1000	6.5344e−2 (8.24e−4) −	6.3989e−2 (5.07e−4) =	6.6852e−1 (7.26e−3) −	4.6015e−1 (1.98e−2) −	6.4019e−2 (3.73e−4) =	7.0392e−2 (2.98e−3) −	6.7060e−2 (2.42e−3) −	**6.3968e−2 (8.77e−4)**
	3	500	2.3400e−1 (4.05e−3) −	2.3455e−1 (4.14e−3) −	8.8108e−1 (2.33e−2) −	4.6447e−1 (1.97e−2) −	2.2810e−1 (5.26e−4) =	2.3837e−1 (5.25e−3) −	2.6659e−1 (4.67e−3) −	**2.0473e−1 (4.35e−2)**
		1000	2.3282e−1 (1.90e−3) −	2.3212e−1 (2.30e−3) −	8.7928e−1 (1.77e−2) −	5.9750e−1 (2.06e−2) −	2.2771e−1 (5.91e−4) =	2.3725e−1 (2.88e−3) −	2.6483e−1 (3.61e−3) −	**2.0536e−1 (4.26e−2)**
WFG6	2	500	1.4080e−2 (4.36e−4) −	1.4401e−2 (5.69e−4) −	8.2397e−1 (4.48e−3) −	1.8586e−1 (1.31e−2) −	3.7565e−2 (7.87e−3) −	1.7044e−2 (2.03e−3) −	1.7685e−1 (5.83e−2) −	**1.1916e−2 (3.57e−3)**
		1000	1.3528e−2 (9.95e−4) −	4.3051e−2 (1.33e−1) −	8.2894e−1 (5.41e−3) −	3.5087e−1 (9.36e−3) −	7.5269e−2 (1.33e−2) −	1.5611e−2 (1.27e−3) −	2.4116e−1 (1.03e−1) −	**1.3511e−2 (1.80e−3)**
	3	500	2.2274e−1 (8.60e−4) =	2.2358e−1 (2.67e−3) =	1.0732e+0 (2.91e−2) −	4.9373e−1 (1.54e−2) −	2.4003e−1 (1.63e−2) −	2.3896e−1 (9.01e−3) −	2.8374e−1 (3.97e−2) −	**1.9537e−1 (5.34e−2)**
		1000	2.2251e−1 (1.14e−3) −	2.2369e−1 (2.57e−3) =	1.0911e+0 (3.23e−2) −	6.4126e−1 (1.80e−2) −	2.7019e−1 (4.06e−2) −	2.3973e−1 (2.78e−2) −	4.0591e−1 (1.46e−1) −	**1.9888e−1 (5.67e−2)**
WFG7	2	500	2.0879e−1 (7.21e−3) −	2.7954e−1 (1.20e−2) −	6.1241e−1 (5.38e−3) −	1.0539e−1 (8.19e−3) −	2.0149e−1 (1.16e−2) −	2.4800e−1 (2.18e−2) −	2.1756e−1 (1.51e−2) −	**7.6683e−2 (8.26e−3)**
		1000	2.4811e−1 (5.22e−3) −	3.0531e−1 (1.06e−2) −	6.1917e−1 (6.28e−3) −	2.1820e−1 (1.75e−2) −	2.4333e−1 (9.73e−3) −	2.7895e−1 (6.43e−3) −	2.5411e−1 (1.19e−2) −	**8.1005e−2 (9.26e−3)**
	3	500	4.0654e−1 (1.55e−2) −	4.3677e−1 (1.89e−2) −	8.5235e−1 (2.69e−2) −	4.2242e−1 (1.00e−2) −	3.5533e−1 (1.20e−2) −	4.1415e−1 (1.68e−2) −	3.5793e−1 (1.29e−2) −	**3.1527e−1 (5.61e−2)**
		1000	4.1818e−1 (9.37e−3) −	4.3783e−1 (2.26e−2) −	8.5379e−1 (3.20e−2) −	5.3300e−1 (2.14e−2) −	3.6947e−1 (1.07e−2) −	4.2586e−1 (1.27e−2) −	3.6609e−1 (1.08e−2) =	**3.3231e−1 (6.06e−2)**
WFG8	2	500	2.0378e−1 (2.92e−2) −	3.0425e−1 (1.50e−2) −	6.4431e−1 (9.42e−3) −	1.5968e−1 (4.84e−3) −	1.3177e−1 (1.95e−2) −	2.6683e−1 (1.87e−2) −	1.9545e−1 (2.05e−2) −	**6.5890e−2 (3.05e−3)**
		1000	1.9119e−1 (2.87e−2) −	3.0034e−1 (2.37e−2) −	6.3820e−1 (7.62e−3) −	2.3198e−1 (7.14e−3) −	1.5688e−1 (1.12e−2) −	2.6195e−1 (3.44e−2) −	2.0671e−1 (1.55e−2) −	**6.5476e−2 (2.62e−3)**
	3	500	4.0182e−1 (1.81e−2) −	4.5363e−1 (1.73e−2) −	8.9668e−1 (2.35e−2) −	4.2102e−1 (8.04e−3) −	2.9013e−1 (5.63e−3) −	4.1335e−1 (8.63e−3) −	4.0052e−1 (1.78e−2) −	**2.3226e−1 (4.19e−2)**
		1000	3.8762e−1 (2.52e−2) −	4.2978e−1 (2.08e−2) −	8.8701e−1 (2.46e−2) −	4.9458e−1 (1.58e−2) −	2.9627e−1 (1.47e−2) −	4.1844e−1 (1.96e−2) −	3.8068e−1 (2.27e−2) −	**2.2806e−1 (4.26e−2)**
WFG9	2	500	1.5924e−2 (1.73e−3) =	1.8613e−2 (9.96e−3) −	8.5105e−1 (1.33e−2) −	1.3459e−1 (1.56e−2) −	1.4955e−1 (1.71e−2) −	3.7209e−2 (1.90e−2) −	1.4274e−1 (5.96e−2) −	**1.4470e−2 (5.39e−3)**
		1000	**1.5699e−2 (1.98e−3) =**	2.0404e−2 (2.59e−2) =	8.5552e−1 (1.17e−2) −	2.7468e−1 (5.34e−2) −	1.6288e−1 (1.39e−2) −	2.9029e−2 (9.00e−3) −	1.6329e−1 (4.58e−2) −	1.8037e−2 (1.06e−2)
	3	500	2.5689e−1 (1.76e−2) −	2.2035e−1 (4.91e−3) −	1.1452e+0 (2.96e−2) −	4.5362e−1 (5.05e−2) −	2.6902e−1 (8.54e−3) −	2.6550e−1 (1.79e−2) −	2.9950e−1 (4.91e−2) −	**2.0585e−1 (4.39e−2)**
		1000	2.5567e−1 (1.70e−2) −	2.3289e−1 (2.79e−2) =	1.1398e+0 (3.26e−2) −	6.2198e−1 (7.53e−2) −	2.6855e−1 (8.92e−3) −	2.6809e−1 (1.57e−2) −	2.8863e−1 (4.45e−2) −	**2.0701e−1 (4.09e−2)**
+/−/=			0/32/4	1/30/5	0/36/0	1/35/0	6/25/5	0/36/0	1/34/1

**Table 10:** The statistical HV results with LSTPA, DGEA, SSCEA, IM-MOEA/D, FDV, ATLMOEA, LERD and MPSLMOCSO on 36 WFG test problems.

Problem	M	D	LSTPA	DGEA	SSCEA	IM-MOEA/D	FDV	ATLMOEA	LERD	MPSLMOCSO
WFG1	2	500	1.0961e−1 (1.21e−2) −	1.1513e−1 (5.27e−2) −	0.0000e+0 (0.00e+0) −	3.0253e−2 (2.91e−2) −	1.1600e−1 (7.40e−2) −	1.1588e−1 (1.55e−2) −	**1.8071e−1 (1.95e−3) =**	1.8086e−1 (4.12e−3)
		1000	1.0264e−1 (1.14e−2) −	1.1549e−1 (4.61e−2) −	0.0000e+0 (0.00e+0) −	4.0356e−3 (1.41e−2) −	6.3443e−2 (4.77e−2) −	1.1587e−1 (1.47e−2) −	**1.7671e−1 (1.44e−3) +**	1.6227e−1 (3.63e−2)
	3	500	2.5640e−1 (1.19e−2) −	2.8289e−1 (9.33e−3) −	0.0000e+0 (0.00e+0) −	1.1946e−1 (2.87e−2) −	2.5585e−1 (7.33e−2) −	2.6556e−1 (9.43e−3) −	2.9844e−1 (6.51e−3) −	**3.4138e−1 (5.29e−2)**
		1000	2.5022e−1 (8.29e−3) −	2.8288e−1 (6.40e−3) −	0.0000e+0 (0.00e+0) −	4.8097e−2 (2.60e−2) −	2.0363e−1 (4.92e−2) −	2.6209e−1 (1.00e−2) −	2.9959e−1 (7.70e−3) −	**3.2870e−1 (1.65e−2)**
WFG2	2	500	5.0069e−1 (8.69e−3) −	4.6576e−1 (1.21e−2) −	3.0497e−1 (5.63e−3) −	5.1437e−1 (5.82e−3) −	5.2544e−1 (5.43e−3) −	4.8870e−1 (1.03e−2) −	5.2818e−1 (1.07e−2) −	**5.7197e−1 (5.50e−3)**
		1000	4.8667e−1 (1.21e−2) −	4.5931e−1 (5.66e−3) −	3.0437e−1 (4.77e−3) −	4.6640e−1 (7.64e−3) −	5.2663e−1 (4.36e−3) −	4.7538e−1 (6.60e−3) −	5.0406e−1 (7.90e−3) −	**5.6993e−1 (6.76e−3)**
	3	500	7.4781e−1 (1.92e−2) −	7.0107e−1 (1.46e−2) −	4.7108e−1 (2.58e−2) −	7.2289e−1 (1.19e−2) −	8.0957e−1 (4.78e−3) =	7.4427e−1 (1.51e−2) −	7.5443e−1 (9.77e−3) −	**8.1070e−1 (6.51e−3)**
		1000	7.3147e−1 (1.70e−2) −	7.0011e−1 (1.23e−2) −	4.7063e−1 (2.19e−2) −	6.7901e−1 (9.48e−3) −	7.8855e−1 (4.70e−3) −	7.3008e−1 (1.44e−2) −	7.3340e−1 (1.17e−2) −	**8.0640e−1 (5.85e−3)**
WFG3	2	500	4.6381e−1 (3.11e−3) −	4.1314e−1 (5.25e−3) −	2.5011e−1 (1.22e−3) −	4.7021e−1 (6.86e−3) −	4.8860e−1 (4.01e−3) −	4.5221e−1 (4.76e−3) −	4.5861e−1 (8.98e−3) −	**5.3532e−1 (4.55e−3)**
		1000	4.4712e−1 (3.42e−3) −	4.0072e−1 (7.60e−3) −	2.4859e−1 (8.46e−4) −	4.2559e−1 (5.92e−3) −	4.8737e−1 (2.34e−3) −	4.4342e−1 (3.19e−3) −	4.4306e−1 (3.61e−3) −	**5.3031e−1 (5.09e−3)**
	3	500	1.9484e−1 (1.54e−2) −	2.0700e−1 (1.07e−2) −	8.6770e−2 (1.53e−3) −	2.0779e−1 (9.15e−3) −	2.2823e−1 (7.67e−3) −	2.0806e−1 (1.03e−2) −	2.6578e−1 (8.06e−3) −	**2.7496e−1 (1.07e−2)**
		1000	1.8313e−1 (1.58e−2) −	2.0068e−1 (1.57e−2) −	8.6738e−2 (1.48e−3) −	1.6371e−1 (9.80e−3) −	2.2357e−1 (8.49e−3) −	2.0208e−1 (1.17e−2) −	2.5741e−1 (8.71e−3) −	**2.7770e−1 (1.20e−2)**
WFG4	2	500	2.7229e−1 (1.91e−3) −	2.4286e−1 (7.62e−3) −	1.3287e−1 (1.17e−3) −	3.0202e−1 (2.40e−3) +	**3.1431e−1 (1.86e−4) +**	2.6105e−1 (1.43e−3) −	2.6883e−1 (8.05e−3) −	2.8836e−1 (2.02e−3)
		1000	2.6441e−1 (1.66e−3) −	2.3805e−1 (6.12e−3) −	1.3161e−1 (1.13e−3) −	2.7316e−1 (3.84e−3) −	**3.0851e−1 (1.23e−3) +**	2.5726e−1 (1.40e−3) −	2.5623e−1 (5.90e−3) −	2.8548e−1 (1.51e−3)
	3	500	4.4083e−1 (3.40e−3) −	4.1496e−1 (7.32e−3) −	2.4700e−1 (7.66e−3) −	4.4196e−1 (5.47e−3) −	**4.9701e−1 (3.11e−3) +**	4.4379e−1 (6.08e−3) −	4.4483e−1 (4.71e−3) −	4.5918e−1 (3.70e−3)
		1000	4.3416e−1 (5.36e−3) −	4.1606e−1 (9.11e−3) −	2.4612e−1 (7.96e−3) −	3.9821e−1 (7.48e−3) −	**4.8727e−1 (3.53e−3) +**	4.3459e−1 (7.15e−3) −	4.4566e−1 (5.60e−3) −	4.5813e−1 (2.91e−3)
WFG5	2	500	3.1143e−1 (1.39e−3) −	3.1272e−1 (1.80e−4) −	7.3123e−2 (7.35e−4) −	1.9453e−1 (6.81e−3) −	**3.1292e−1 (3.12e−5) +**	3.0875e−1 (1.83e−3) −	3.1040e−1 (2.06e−3) −	3.1206e−1 (6.81e−4)
		1000	3.1127e−1 (8.68e−4) −	3.1266e−1 (3.19e−4) =	7.2770e−2 (7.08e−4) −	1.2086e−1 (6.83e−3) −	3.1295e−1 (2.82e−5) =	3.0920e−1 (1.46e−3) −	3.1054e−1 (2.28e−3) −	**3.1310e−1 (1.65e−3)**
	3	500	4.9494e−1 (5.56e−3) −	4.9844e−1 (5.42e−3) −	1.8072e−1 (3.36e−3) −	3.4718e−1 (1.51e−2) −	**5.1219e−1 (4.31e−4) +**	4.9341e−1 (4.76e−3) −	4.8102e−1 (4.95e−3) −	5.0841e−1 (1.26e−2)
		1000	4.9645e−1 (3.38e−3) −	5.0275e−1 (4.06e−3) =	1.8096e−1 (2.52e−3) −	2.6357e−1 (1.35e−2) −	**5.1247e−1 (4.95e−4) +**	4.9423e−1 (4.12e−3) −	4.8266e−1 (3.84e−3) −	5.0189e−1 (2.91e−3)
WFG6	2	500	3.4428e−1 (3.65e−4) =	3.4427e−1 (6.37e−4) −	3.9206e−2 (7.29e−4) −	2.4536e−1 (6.72e−3) −	3.2804e−1 (4.65e−3) −	3.4296e−1 (6.58e−4) −	2.5294e−1 (3.24e−2) −	**3.4494e−1 (1.43e−3)**
		1000	3.4543e−1 (6.82e−4) −	3.3682e−1 (3.80e−2) =	3.8275e−2 (6.83e−4) −	1.6471e−1 (4.28e−3) −	3.0659e−1 (7.32e−3) −	3.4448e−1 (3.46e−4) −	2.2183e−1 (5.46e−2) −	**3.4597e−1 (1.42e−3)**
	3	500	5.4884e−1 (1.71e−3) −	5.4732e−1 (2.63e−3) =	1.2678e−1 (2.91e−3) −	3.3943e−1 (1.26e−2) −	5.0910e−1 (3.49e−2) −	5.3807e−1 (4.66e−3) −	5.1671e−1 (4.49e−2) −	**5.5151e−1 (1.26e−2)**
		1000	5.4993e−1 (1.79e−3) =	5.4768e−1 (3.04e−3) =	1.2641e−1 (2.56e−3) −	2.4765e−1 (1.00e−2) −	4.8143e−1 (5.94e−2) −	5.4262e−1 (4.60e−3) −	5.0628e−1 (5.54e−2) −	**5.5088e−1 (1.47e−2)**
WFG7	2	500	2.3500e−1 (3.66e−3) −	1.9973e−1 (5.51e−3) −	8.0135e−2 (1.45e−3) −	2.8965e−1 (4.26e−3) −	2.3908e−1 (6.06e−3) −	2.1391e−1 (1.13e−2) −	2.3141e−1 (7.58e−3) −	**3.0353e−1 (2.55e−3)**
		1000	2.1464e−1 (2.48e−3) −	1.8833e−1 (4.56e−3) −	8.0178e−2 (6.97e−4) −	2.2824e−1 (9.00e−3) −	2.1720e−1 (4.99e−3) −	1.9959e−1 (2.28e−3) −	2.1231e−1 (6.05e−3) −	**3.0445e−1 (5.51e−3)**
	3	500	3.6716e−1 (9.37e−3) −	3.4754e−1 (1.03e−2) −	1.9365e−1 (4.53e−3) −	4.0669e−1 (6.33e−3) =	3.9795e−1 (8.10e−3) −	3.6249e−1 (1.06e−2) −	4.1085e−1 (9.75e−3) =	**4.1666e−1 (1.57e−2)**
		1000	3.5905e−1 (5.23e−3) −	3.4681e−1 (1.21e−2) −	1.9435e−1 (3.83e−3) −	3.1970e−1 (1.60e−2) −	3.8751e−1 (6.95e−3) −	3.5478e−1 (7.37e−3) −	4.0439e−1 (8.24e−3) =	**4.1393e−1 (3.46e−2)**
WFG8	2	500	2.3886e−1 (1.51e−2) −	1.8918e−1 (6.74e−3) −	7.3525e−2 (1.50e−3) −	2.6028e−1 (2.63e−3) −	2.7588e−1 (1.05e−2) −	2.0604e−1 (9.14e−3) −	2.4289e−1 (1.07e−2) −	**3.1201e−1 (1.73e−3)**
		1000	2.4503e−1 (1.48e−2) −	1.9095e−1 (1.07e−2) −	7.4313e−2 (1.68e−3) −	2.2088e−1 (3.46e−3) −	2.6241e−1 (5.95e−3) −	2.0877e−1 (1.75e−2) −	2.3687e−1 (8.06e−3) −	**3.1143e−1 (1.16e−3)**
	3	500	3.7134e−1 (1.17e−2) −	3.3902e−1 (8.55e−3) −	1.7649e−1 (4.80e−3) −	3.9108e−1 (6.32e−3) −	4.4394e−1 (4.62e−3) −	3.6405e−1 (4.76e−3) −	3.8069e−1 (1.00e−2) −	**4.8765e−1 (1.57e−2)**
		1000	3.7983e−1 (1.57e−2) −	3.5199e−1 (1.25e−2) −	1.8146e−1 (3.62e−3) −	3.3488e−1 (7.98e−3) −	4.3890e−1 (1.11e−2) −	3.6184e−1 (1.21e−2) −	3.9365e−1 (1.54e−2) −	**4.7967e−1 (5.05e−3)**
WFG9	2	500	**3.4299e−1 (8.05e−4) =**	3.4137e−1 (6.12e−3) =	3.5271e−2 (2.83e−3) −	2.7458e−1 (8.32e−3) −	2.6601e−1 (8.94e−3) −	3.3465e−1 (6.39e−3) −	2.7118e−1 (3.34e−2) −	3.4254e−1 (3.40e−3)
		1000	**3.4353e−1 (1.50e−3) =**	3.4138e−1 (1.51e−2) =	3.5494e−2 (1.83e−3) −	2.0273e−1 (2.54e−2) −	2.5899e−1 (7.32e−3) −	3.3702e−1 (3.77e−3) =	2.5981e−1 (2.53e−2) −	3.4020e−1 (7.09e−3)
	3	500	4.7967e−1 (2.48e−2) −	**5.3883e−1 (7.84e−3) +**	1.0737e−1 (4.92e−3) −	3.5786e−1 (3.58e−2) −	4.5804e−1 (8.06e−3) −	4.8621e−1 (2.40e−2) −	4.6839e−1 (4.06e−2) −	5.1346e−1 (8.23e−3)
		1000	4.7754e−1 (2.10e−2) −	**5.2608e−1 (2.38e−2) +**	1.1040e−1 (2.52e−3) −	2.5303e−1 (3.88e−2) −	4.5965e−1 (8.32e−3) −	4.7153e−1 (2.26e−2) −	4.7430e−1 (4.22e−2) −	5.2022e−1 (8.11e−3)
+/−/=			0/32/4	2/27/7	0/36/0	1/35/1	7/27/2	0/35/1	1/32/3

**Table 11:** The statistical IGD results with LSTPA, DGEA, SSCEA, IM-MOEA/D, FDV ATLMOEA, LERD and MPSLMOCSO on 20 UF test problems.

Problem	M	D	LSTPA	DGEA	SSCEA	IM-MOEA/D	FDV	ATLMOEA	LERD	MPSLMOCSO
UF1	2	500	3.6471e−1 (1.24e−1) −	3.2409e−1 (4.45e−2) −	2.0964e+0 (4.82e−2) −	7.8115e−1 (1.08e−1) −	4.1301e−1 (2.37e−1) =	3.4884e−1 (1.72e−2) −	6.4016e−1 (6.92e−2) −	**1.8896e−1 (2.07e−2)**
		1000	4.2135e−1 (1.27e−1) −	3.5429e−1 (6.30e−2) −	2.1746e+0 (2.59e−2) −	1.2935e+0 (7.78e−2) −	4.4239e−1 (2.23e−1) =	3.6080e−1 (1.18e−2) −	1.0105e+0 (5.10e−2) −	**2.2823e−1 (1.47e−2)**
UF2	2	500	1.4771e−1 (9.48e−3) −	2.5278e−1 (1.62e−2) −	9.2267e−1 (1.52e−2) −	2.9710e−1 (5.38e−2) −	1.5695e−1 (2.00e−2) −	1.5594e−1 (9.60e−3) −	1.2242e−1 (4.46e−3) −	**8.1759e−2 (7.27e−4)**
		1000	1.7520e−1 (7.54e−3) −	3.0114e−1 (4.17e−2) −	9.4502e−1 (8.12e−3) −	4.3447e−1 (3.82e−2) −	2.0153e−1 (1.88e−2) −	1.6393e−1 (7.44e−3) −	1.3672e−1 (8.20e−3) −	**8.3504e−2 (4.56e−4)**
UF3	2	500	1.2178e−1 (1.11e−1) −	1.2994e−1 (2.22e−3) −	1.1138e+0 (2.64e−2) −	3.9481e−1 (1.50e−2) −	2.0074e−1 (2.02e−1) −	1.2660e−1 (2.36e−3) −	2.6353e−1 (1.32e−2) −	**3.3198e−2 (2.69e−3)**
		1000	1.1258e−1 (4.32e−3) −	1.3842e−1 (6.13e−2) −	1.1238e+0 (1.72e−2) −	6.6551e−1 (2.86e−2) −	3.0868e−1 (2.25e−1) −	1.2435e−1 (1.86e−3) −	3.5001e−1 (3.65e−2) −	**2.3900e−2 (2.69e−3)**
UF4	2	500	1.2874e−1 (1.30e−2) −	1.2500e−1 (1.74e−2) −	2.2247e−1 (1.53e−3) −	1.6200e−1 (2.91e−3) −	1.2506e−1 (8.84e−3) −	1.1381e−1 (8.32e−3) −	1.2139e−1 (2.05e−3) −	**4.9765e−2 (2.40e−3)**
		1000	1.2801e−1 (1.55e−2) −	1.3455e−1 (1.43e−3) −	2.2444e−1 (1.18e−3) −	1.8477e−1 (3.24e−3) −	1.2756e−1 (1.08e−2) −	1.1355e−1 (9.69e−3) −	1.2694e−1 (1.84e−3) −	**4.9182e−2 (2.66e−3)**
UF5	2	500	1.5502e+0 (3.74e−1) +	3.8582e+0 (8.49e−1) −	7.0799e+0 (1.01e−1) −	3.1842e+0 (1.21e−1) −	**9.6862e−1 (3.68e−2) +**	2.6674e+0 (2.25e−1) −	2.3335e+0 (1.40e−1) −	1.6203e+0 (2.21e−1)
		1000	2.3783e+0 (5.04e−1) =	4.6139e+0 (1.04e+0) −	7.2220e+0 (1.09e−1) −	4.3760e+0 (1.53e−1) −	**1.1544e+0 (3.97e−2) +**	3.1833e+0 (1.24e−1) −	3.0286e+0 (5.74e−1) −	2.1621e+0 (2.16e−1)
UF6	2	500	7.0377e−1 (3.69e−1) =	1.0985e+0 (9.56e−2) −	8.4499e+0 (1.83e−1) −	2.2931e+0 (2.03e−1) −	**5.2885e−1 (1.84e−3) +**	1.2074e+0 (2.08e−1) −	1.3035e+0 (4.61e−1) −	6.0570e−1 (9.06e−2)
		1000	8.3993e−1 (2.69e−1) +	1.5297e+0 (9.66e−2) −	8.7464e+0 (1.44e−1) −	4.9992e+0 (2.60e−1) −	**5.2182e−1 (3.68e−2) +**	1.4760e+0 (2.16e−1) −	2.8491e+0 (9.93e−1) −	8.5470e−1 (7.72e−2)
UF7	2	500	6.0464e−1 (1.71e−1) −	3.9297e−1 (1.63e−1) −	2.1561e+0 (3.58e−2) −	8.4941e−1 (1.28e−1) −	3.0483e−1 (3.03e−1) =	3.5640e−1 (2.10e−2) −	6.0466e−1 (5.27e−2) −	**2.3797e−1 (1.87e−2)**
		1000	6.4140e−1 (1.55e−1) −	5.3540e−1 (1.62e−1) −	2.2161e+0 (2.97e−2) −	1.3564e+0 (6.04e−2) −	4.1372e−1 (2.71e−1) =	3.9623e−1 (3.47e−2) −	9.6955e−1 (3.75e−2) −	**2.6317e−1 (4.22e−2)**
UF8	3	500	4.8774e−1 (3.23e−2) =	9.9476e−1 (1.43e−1) −	4.3139e+0 (8.86e−2) −	6.6530e−1 (2.32e−1) −	6.0433e−1 (2.01e−1) −	4.9590e−1 (2.20e−2) −	5.1755e−1 (1.04e−2) −	**4.2937e−1 (7.52e−2)**
		1000	6.5255e−1 (6.24e−2) −	1.0455e+0 (1.93e−1) −	4.4005e+0 (6.31e−2) −	1.5761e+0 (3.95e−1) −	7.4795e−1 (1.40e−1) −	5.2933e−1 (1.66e−2) −	5.2178e−1 (1.33e−2) −	**4.3594e−1 (7.26e−2)**
UF9	3	500	6.8413e−1 (9.41e−2) −	1.1869e+0 (1.40e−1) −	4.4209e+0 (9.99e−2) −	1.6424e+0 (1.79e−1) −	5.4057e−1 (1.61e−1) −	6.9377e−1 (1.82e−2) −	6.2434e−1 (1.90e−2) −	**5.3197e−1 (4.88e−2)**
		1000	8.4324e−1 (1.01e−1) −	1.2828e+0 (1.56e−1) −	4.5114e+0 (7.71e−2) −	2.7642e+0 (2.32e−1) −	7.7408e−1 (8.49e−2) −	7.6066e−1 (1.50e−2) −	6.9889e−1 (1.20e−2) −	**5.2918e−1 (6.17e−2)**
UF10	3	500	4.2127e+0 (4.39e−1) −	6.6139e+0 (9.98e−1) −	2.0786e+1 (4.26e−1) −	9.4541e+0 (1.09e+0) −	2.1742e+0 (5.30e−1) −	3.3602e+0 (2.48e−1) −	1.0262e+0 (1.47e−1) =	**9.9210e−1 (5.14e−1)**
		1000	6.0999e+0 (1.05e+0) −	8.2188e+0 (9.15e−1) −	2.1100e+1 (3.07e−1) −	1.3306e+1 (9.35e−1) −	4.7271e+0 (1.42e+0) −	3.6087e+0 (1.97e−1) −	1.1445e+0 (2.07e−1) −	**1.0137e+0 (4.86e−1)**
+/−/=			2/15/3	0/20/0	0/20/0	0/20/0	4/12/4	0/20/0	0/19/1

**Table 12:** The statistical HV results with LSTPA, DGEA, SSCEA, IM-MOEA/D, FDV, ATLMOEA, LERD and MPSLMOCSO on 20 UF test problems.

Problem	M	D	LSTPA	DGEA	SSCEA	IM-MOEA/D	FDV	ATLMOEA	LERD	MPSLMOCSO
UF1	2	500	2.4613e−1 (9.15e−2) −	2.8032e−1 (3.99e−2) −	0.0000e+0 (0.00e+0) −	4.0856e−2 (3.07e−2) −	2.6567e−1 (2.20e−1) =	2.8027e−1 (1.52e−2) −	9.9049e−2 (3.75e−2) −	**4.4601e−1 (2.70e−2)**
		1000	1.8993e−1 (1.05e−1) −	2.4983e−1 (5.77e−2) −	0.0000e+0 (0.00e+0) −	0.0000e+0 (0.00e+0) −	2.1323e−1 (1.85e−1) =	2.6963e−1 (9.87e−3) −	9.3702e−4 (2.10e−3) −	**3.9620e−1 (1.59e−2)**
UF2	2	500	5.5669e−1 (1.06e−2) −	4.3655e−1 (1.84e−2) −	2.9026e−3 (1.77e−3) −	4.0601e−1 (4.39e−2) −	5.2951e−1 (2.52e−2) −	5.5104e−1 (1.02e−2) −	5.9182e−1 (3.29e−3) −	**6.3283e−1 (8.70e−4)**
		1000	5.2790e−1 (9.16e−3) −	3.8833e−1 (4.37e−2) −	8.1546e−4 (7.94e−4) −	2.6017e−1 (2.57e−2) −	4.8009e−1 (2.25e−2) −	5.4219e−1 (8.46e−3) −	5.7557e−1 (7.63e−3) −	**6.3122e−1 (5.99e−4)**
UF3	2	500	5.8658e−1 (1.16e−1) −	5.7489e−1 (2.56e−3) −	0.0000e+0 (0.00e+0) −	2.2575e−1 (1.36e−2) −	4.7882e−1 (2.46e−1) −	5.7934e−1 (2.70e−3) −	3.6563e−1 (2.27e−2) −	**6.8431e−1 (3.07e−3)**
		1000	5.9563e−1 (6.03e−3) −	5.6257e−1 (8.62e−2) −	0.0000e+0 (0.00e+0) −	5.6775e−2 (1.05e−2) −	3.6883e−1 (2.51e−1) −	5.8270e−1 (2.08e−3) −	2.7691e−1 (3.70e−2) −	**6.9561e−1 (3.09e−3)**
UF4	2	500	2.7736e−1 (1.41e−2) −	2.8050e−1 (1.83e−2) −	1.7463e−1 (9.15e−4) −	2.2980e−1 (1.85e−3) −	2.8093e−1 (9.33e−3) −	2.9086e−1 (1.02e−2) −	2.8643e−1 (2.38e−3) −	**3.7900e−1 (3.24e−3)**
		1000	2.7819e−1 (1.64e−2) −	2.7053e−1 (1.43e−3) −	1.7377e−1 (8.27e−4) −	2.0515e−1 (1.81e−3) −	2.7857e−1 (1.10e−2) −	2.9113e−1 (1.14e−2) −	2.7984e−1 (2.32e−3) −	**3.7940e−1 (3.44e−3)**
UF5	2	500	0.0000e+0 (0.00e+0) =	0.0000e+0 (0.00e+0) =	0.0000e+0 (0.00e+0) =	0.0000e+0 (0.00e+0) =	0.0000e+0 (0.00e+0) =	0.0000e+0 (0.00e+0) =	0.0000e+0 (0.00e+0) =	0.0000e+0 (0.00e+0)
		1000	0.0000e+0 (0.00e+0) =	0.0000e+0 (0.00e+0) =	0.0000e+0 (0.00e+0) =	0.0000e+0 (0.00e+0) =	0.0000e+0 (0.00e+0) =	0.0000e+0 (0.00e+0) =	0.0000e+0 (0.00e+0) =	0.0000e+0 (0.00e+0)
UF6	2	500	1.2048e−2 (1.06e−2) =	0.0000e+0 (0.00e+0) −	0.0000e+0 (0.00e+0) −	0.0000e+0 (0.00e+0) −	**8.5207e−2 (1.18e−3) +**	0.0000e+0 (0.00e+0) −	3.3481e−2 (8.16e−2) +	1.5680e−2 (1.31e−2)
		1000	0.0000e+0 (0.00e+0) −	0.0000e+0 (0.00e+0) −	0.0000e+0 (0.00e+0) −	0.0000e+0 (0.00e+0) −	**7.5852e−2 (8.32e−3) +**	0.0000e+0 (0.00e+0) −	1.3211e−2 (4.24e−2) =	1.0819e−3 (4.80e−3)
UF7	2	500	1.0226e−1 (6.52e−2) −	1.5829e−1 (7.73e−2) −	0.0000e+0 (0.00e+0) −	1.7840e−3 (4.62e−3) −	**3.2903e−1 (1.79e−1) =**	1.5449e−1 (1.97e−2) −	3.7103e−2 (1.91e−2) −	2.7246e−1 (2.03e−2)
		1000	4.5639e−2 (4.72e−2) −	7.6122e−2 (7.00e−2) −	0.0000e+0 (0.00e+0) −	0.0000e+0 (0.00e+0) −	2.1993e−1 (1.23e−1) =	1.2428e−1 (3.04e−2) −	0.0000e+0 (0.00e+0) −	**2.4335e−1 (4.86e−2)**
UF8	3	500	7.2092e−2 (2.81e−2) −	2.0895e−5 (9.34e−5) −	0.0000e+0 (0.00e+0) −	2.6003e−2 (3.12e−2) −	9.8068e−2 (4.80e−2) −	1.0450e−1 (2.07e−2) −	2.8814e−1 (1.44e−2) −	**3.3550e−1 (3.61e−3)**
		1000	7.5342e−3 (8.49e−3) −	0.0000e+0 (0.00e+0) −	0.0000e+0 (0.00e+0) −	0.0000e+0 (0.00e+0) −	1.2068e−2 (2.30e−2) −	7.5988e−2 (1.52e−2) −	2.5267e−1 (3.38e−2) −	**3.2728e−1 (2.12e−2)**
UF9	3	500	1.3519e−1 (5.51e−2) −	1.1595e−3 (5.19e−3) −	0.0000e+0 (0.00e+0) −	0.0000e+0 (0.00e+0) −	2.9005e−1 (7.47e−2) =	1.1789e−1 (1.22e−2) −	2.1049e−1 (1.10e−2) −	**2.9285e−1 (3.47e−2)**
		1000	4.0430e−2 (4.18e−2) −	1.5739e−3 (5.03e−3) −	0.0000e+0 (0.00e+0) −	0.0000e+0 (0.00e+0) −	8.3674e−2 (3.91e−2) −	6.4479e−2 (1.20e−2) −	1.5637e−1 (7.74e−3) −	**2.8409e−1 (2.96e−2)**
UF10	3	500	0.0000e+0 (0.00e+0) −	0.0000e+0 (0.00e+0) −	0.0000e+0 (0.00e+0) −	0.0000e+0 (0.00e+0) −	0.0000e+0 (0.00e+0) −	0.0000e+0 (0.00e+0) −	0.0000e+0 (0.00e+0) −	**3.8164e−2 (4.62e−2)**
		1000	0.0000e+0 (0.00e+0) −	0.0000e+0 (0.00e+0) −	0.0000e+0 (0.00e+0) −	0.0000e+0 (0.00e+0) −	0.0000e+0 (0.00e+0) −	0.0000e+0 (0.00e+0) −	0.0000e+0 (0.00e+0) −	**2.4809e−2 (3.79e−2)**
+/−/=			0/17/3	0/18/2	0/18/2	0/18/2	2/11/7	0/18/2	1/16/3	

**Figure 5:**
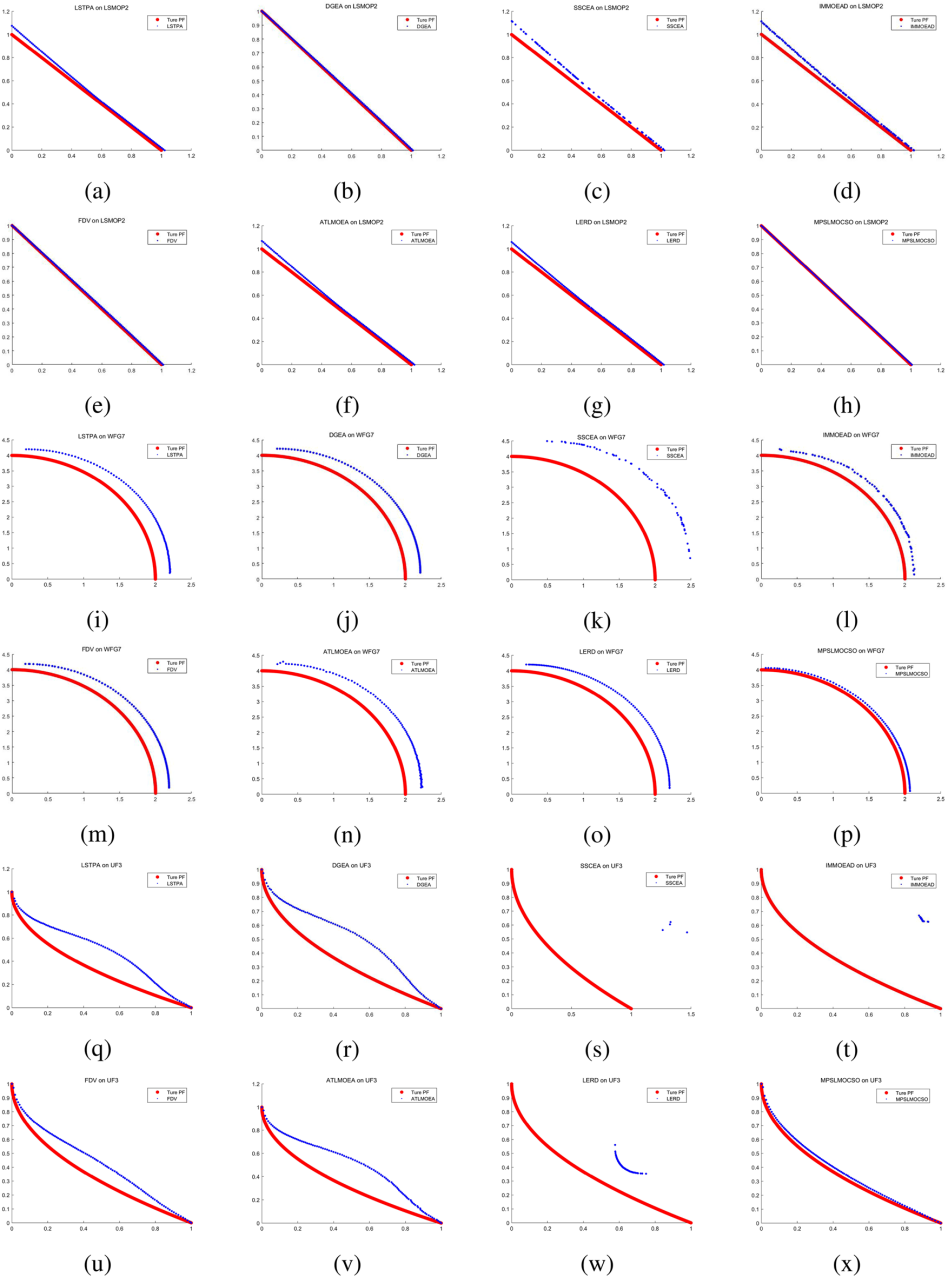
Pareto optimal solutions obtained from the best IGD values of eight algorithms running on the LSMOP2, WFG7 and UF3 with 2 objectives and 1000 decision variables.

**Figure 6:**
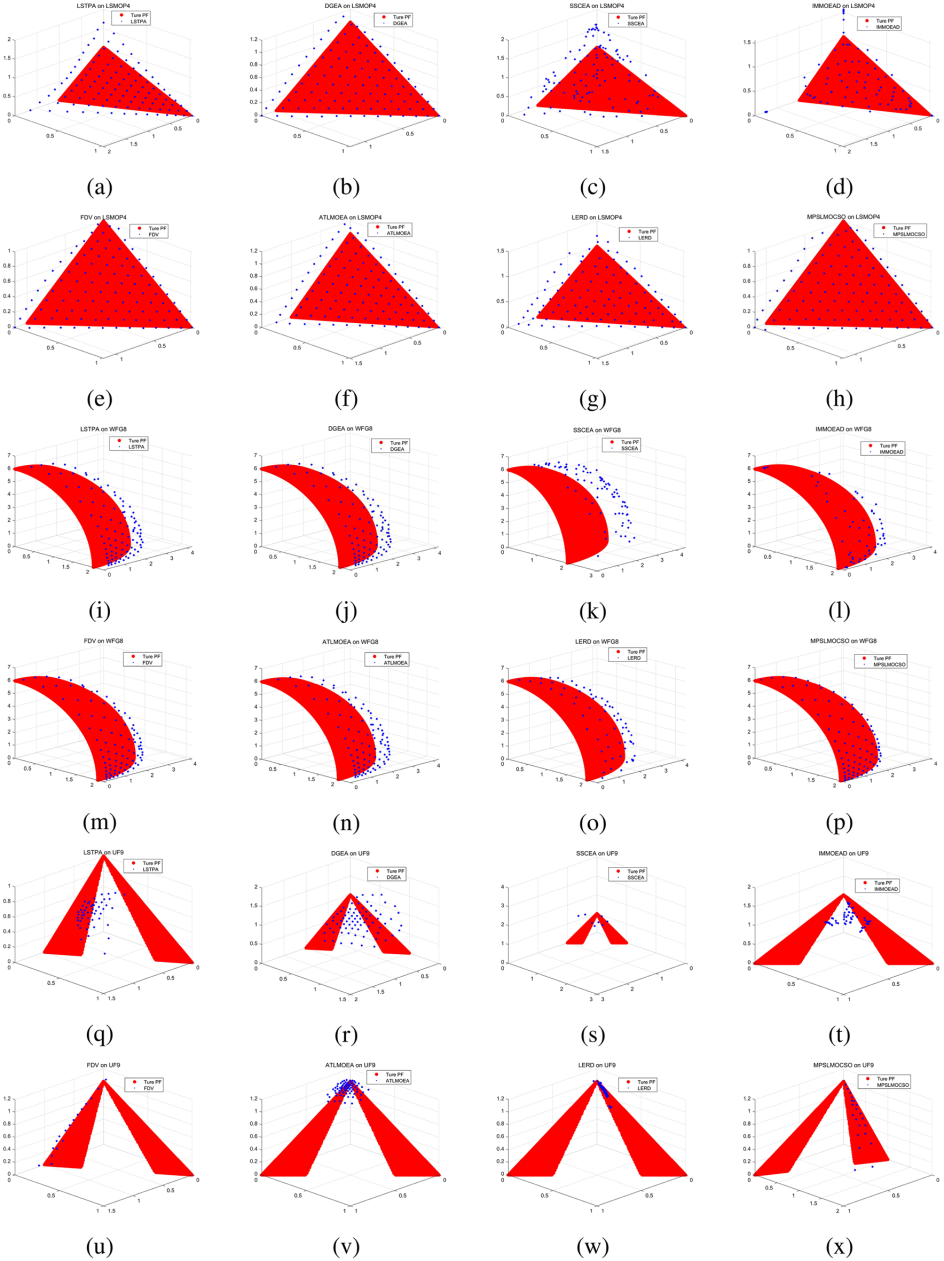
Pareto optimal solutions obtained from the best IGD values of eight algorithms running on the LSMOP4, WFG8 and UF9 with 3 objectives and 500 decision variables.

The lower triangle, circle, upper triangle, hexagon, square, plus, asterisk and diamond symbols respectively denote the optimal average IGD of LSTPA, DGEA, SSCEA, IM-MOEA/D, FDV, ATLMOEA, LERD and LMOCSO achieved for the corresponding test problems, while the horizontal axis denotes the corresponding test problem. For example, in Fig. 4b , the “$\bigcirc$” indicated that DGEA gets the optimal average IGD for WFG9 among the eight algorithms. The “$\Box$” indicates that FDV derives the optimal average IGDs for WFG2, WFG4, WFG5 and WFG8 compared to other seven algorithms. For LSMOP1-LSMOP9, LMOCSO achieves the best results only on LSMOP9, while FDV and ATLMOEA achieve 4 and 4 best results. For the other two test suites, LMOCSO fails to achieve the optimal results, which suggests that LMOCSO does not exhibit significant advantages in performance.

The following experiments are conducted on three function sets using LMOCSO as the optimizer in MPSOF. The resulting average IGD and HV values are depicted in Tables 7 and 8.

Table 7 displays that MPSLMOCSO has the best average performance for large part of instances among the compared algorithms. Notably, it achieves the optimal outcomes for LSMOP1, LSMOP4, LSMOP6, and LSMOP7. This is attributed to MPSLMOCSO combining problem transformation mechanism with a multi-population method, enabling multiple populations to search within a small-scale space and achieve a balance between diversity and convergence. The performance of LSTPA, DGEA, SSCEA, IM-MOEA/D, ARMOEA and LERD is subpar compared to MPSLMOCSO, probably because LSTPA is fundamentally accelerated by CSO, which may not be sufficiently effective in searching within the limited evaluation numbers in the context of complex and high-dimensional problems. DGEA is not able to accurately determine the parental populations when searching for decision spaces with similar information, resulting in the algorithm being trapped in a local optimum. MPSLMOCSO performs less well than FDV on the LSMOP9 problem. From the results in the last row, LSTPA, SSCEA and LERD perform worse than MPSLMOCSO on 36 test instances. DGEA performs worse than MPSLMOCSO in 31 test instances, and its performance is similar to the algorithm in this paper on 5 test instances. While IM-MOEA/D performs worse than and similar to MPSLMOCSO in 35 and 1 test instances. FDV performs better than MPSLMOCSO in 6 test instances, but falls short of MPSLMOCSO in 27 test instances, and exhibits comparable performance to MPSLMOCSO in 3 test instances. ATLMOEA shows similar performance to MPSLMOCSO across 32 test instances, but is inferior to MPSLMOCSO in 4 instances.

**Figure 7:**
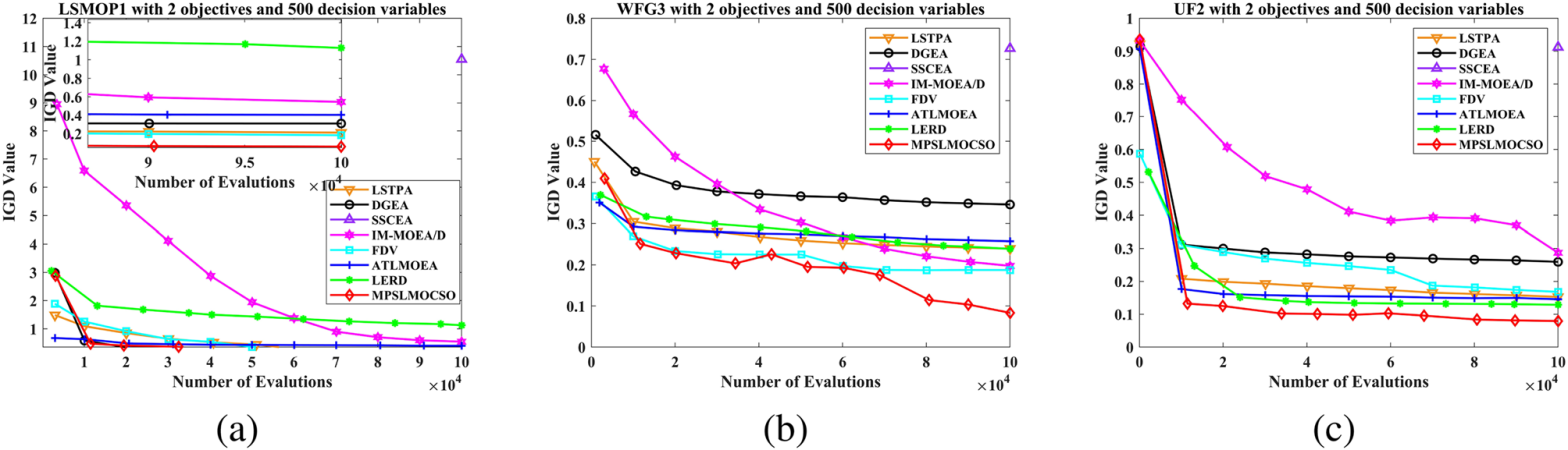
The convergence curves of IGD results for LSTPA, DGEA, SSCEA, IM-MOEA/D, FDV, ATLMOEA, LERD and MPSLMOCSO.

Table 8 displays that MPSLMOCSO excels in achieving superior performance in 26 out of the 36 test instances. All algorithms yield an HV value of zero for LSMOP3 with 2 objectives and decision variables of 500 and 1000 dimensions. The same is true for the 2-objective LSMOP7. This indicates that none of them could generate any satisfactory solutions in the final generation. Table 8 also indicates that LSTPA, SSCEA and LERD perform similarly to MPSLMOCSO on 4 test instances, both IM-MOEA/D and ATLMOEA perform similarly to MPSLMOCSO on 5 test instances, while DGEA and FDV algorithms perform similarly to MPSLMOCSO 6 test problems. LSTPA, SSCEA and LERD are less effective than MPSLMOCSO on 32 test instances, IM-MOEA/D is less effective than MPSLMOCSO on 31 test instances, both DGEA and ATLMOEA are inferior to MPSLMOCSO on 30 test instances. FDV is inferior to MPSLMOCSO on 27 test problems. FDV and ATLMOEA are superior to MPSLMOCSO on 3 and 1 test instances, respectively.

Tables 9 and 10 depict the statistical outcomes of IGD and HV values of different algorithms on WFG.

Table 9 indicates that MPSLMOCSO is more competitive on WFG compared to other algorithms, achieving 29 best solutions in terms of average IGD out of 36 test cases. LSTPA performs better on WFG9 with 2 objectives and 1000-dimensional decision variables. It is evident that the IGD values of MPSLMOCSO and LSTPA do not differ significantly. FDV mainly excels on WFG4. The last row of the table shows that SSCEA and ATLMOEA are inferior to MPSLMOCSO on 36 test instances. This is primarily due to the fact that SSCEA relies on DVA for variable classification, which may result in imprecise classification in complex high-dimensional search spaces when using perturbation methods. ATLMOEA uses neural network training for information retrieval, but the accuracy of information from limited training sessions cannot be guaranteed. DGEA outperforms MPSLMOCSO on 1 test instance, lags behind on 30 test instances, and performs similarly on 5 instances. IM-MOEA/D outperforms MPSLMOCSO on only 1 instance and is inferior to MPSLMOCSO on 35 test instances. FDV and LERD outperform MPSLMOCSO on 6 and 1 test instances respectively, but fall short on 25 and 34 test instances, and perform similarly on 5 and 1 instances respectively.

Table 10 shows that MPSLMOCSO achieves 23 best solutions in terms of average HV out of 36 test cases. Additionally, MPSLMOCSO performs well on WFG2, WFG3, WFG7 and WFG8. SSCEA does not achieve optimal values on all test cases, as the adopted method of variable analysis makes it difficult to correctly classify variables, especially when tackling intricate problems involving high-dimensional variables. According to the test results, the performance of LSTPA, DGEA, SSCEA, IM-MOEA/D FDV, ATLMOEA and LERD is lower than that of MPSLMOCSO in 32, 27, 36, 35, 27, 35 and 32 test cases respectively. DGEA, IM-MOEA/D, FDV and LERD exhibit better performance than MPSLMOCSO in 2, 1, 7 and 1 cases, respectively. Furthermore, DGEA, FVD and LERD perform similarly to MPSLMOCSO on 7, 2 and 3 instances, while both IM-MOEA/D and ATLMOEA perform similarly on 1 test instance.

Tables 11 and 12 show the statistical outcomes of IGD and HV values of different algorithms on UF.

Table 11 demonstrates that among the several comparative algorithms, MPSLMOCSO exhibits better comprehensive performance and excels in achieving superior performance in 15 out of 20 cases. FDV exhibits outstanding performance on UF5 and UF6. The test results show that DGEA, SSCEA, IM-MOEA/D and ATLMOEA are all inferior to MPSLMOCSO on 20 test instances. This is attributed to MPSOF enhances population diversity by employing a multi-population approach, and through the optimization design of the second stage, effectively balances convergence and diversity while reducing the likelihood of encountering local optima. LSTPA and FDV outperforms MPSLMOCSO on 2 and 4 test instances, are inferior to MPSLMOCSO on 15 and 12 instances, and are similar to MPSLMOCSO on 3 and 4 instances respectively. LERD is inferior to MPSLMOCSO on 19 instances and similar to MPSLMOCSO on 1 instance.

Table 12 demonstrates that the HV of the eight algorithms is all zero on UF5, indicating that they failed to find any valid solutions for UF5. Out of 20 test problems, MPSLMOCSO achieves optimal values in 14 cases, while FDV achieves optimal values in 3. Across a large portion of the test instances, the performance of LSTPA, DGEA, SSCEA, IM-MOEA/D and ATLMOEA is consistently inferior to the proposed algorithm. Additionally, FDV and LERD outperform MPSLMOCSO on 2 and 1 test cases, underperform MPSLMOCSO on 11 and 16 test cases, and show comparable performance to MPSLMOCSO on 7 and 3 test cases respectively.

In order to visually present the algorithms’ performance more intuitively, this paper chooses two functions (containing different PF shapes) in each test suite and shows the optimized PF for each algorithm in Figs. 5 and 6. The red line denotes the true PF used for comparison, while the blue line denotes the approximate PF generated by algorithms. As depicted in Fig. 5, on the 2-objective problems, MPSLMOCSO exhibits excellent performance both in convergence and distribution. LSTPA, DGEA, FDV, and ATLMOEA produce uniformly distributed but non-convergent solution sets. SSCEA, IM-MOEA/D, and LERD perform poorly on UF3 as they fall into local optima. Figure 6 shows that only MPSLMOCSO obtains solution sets that more uniformly cover the true PFs on LSMOP4 and WFG8. DGEA and FDV exhibit marginally lower performance compared to MPSLMOCSO, while the other algorithms exhibit poorer distribution and convergence. The optimal PFs achieved by MPSLMOCSO and FDV on UF9 do not fully encompass the true Pareto front, yet they still possess a notable advantage over the other algorithms, which are entirely trapped in local optima.

**Figure 8:**
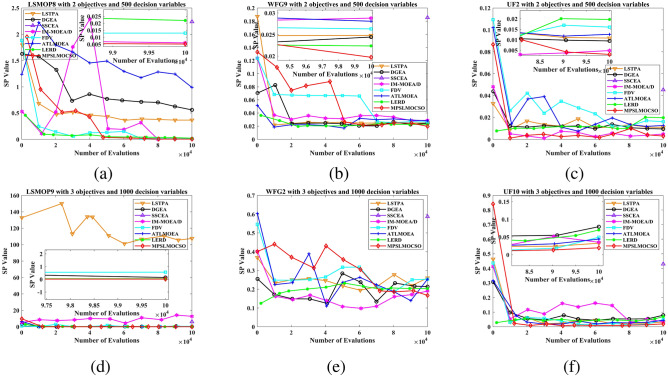
The convergence curves of SP results for LSTPA, DGEA, SSCEA, IM-MOEA/D, FDV, ATLMOEA, LERD and MPSLMOCSO.

Furthermore, the convergence curves of IGD results for eight large-scale MOEAs on different test sets are plotted in Fig. 7. It is obvious that the convergence speed of IM-MOEA/D is slower compared to other algorithms, and it fails to reach a satisfactory level at the end of evolution. FDV and LERD, which are respectively based on problem transformation and reconstruction-based DVA, exhibit faster convergence than MPSLMOCSO in the early stages of evolution. However, their convergence slows down significantly compared to MPSLMOCSO in the later stages, resulting in MPSLMOCSO outperforming them at the end of evolution. MPSLMOCSO exhibits rapid convergence in the early stages, with a slower convergence rate in the mid-term, emphasizing a balance between diversity and convergence, and further converges in the later stages. Moreover, MPSLMOCSO demonstrates superiority across these problems, thereby validating the effectiveness of the proposed three-stage algorithm framework. The convergence curves of SP results of eight algorithms on three different test sets are plotted in Fig. 8, including representative problems of different types of 2-objective and 3-objective in each test set. It illustrates that at the outset of evolution, the diversity of MPSLMOCSO is inferior to that of IM-MOEA/D, which is based on inverse model decomposition, FDV, and LERD. However, in the mid-term of evolution, the SP value declines rapidly, indicating an increase in population diversity. This also demonstrates the effectiveness of the proposed multi-population approach and the hybrid weight updating strategy. At the end of evolution, MPSLMOCSO outperforms other algorithms.

### Real-life application

The precision of voltage transformers’ (VTs) ratio error (RE) is essential for detecting, controlling, and protecting power systems. Therefore, VT manufacturers and users typically conduct regular calibrations and checks to ensure that their performance meets the necessary requirements. The time−varying renewable energy (TREE) estimation problem is transformed into LSMOPs in the paper^40^, enabling real-world issues to be extracted as bi-objective benchmark problems. The effectiveness of MPSOF in practical applications is verified by testing the TREE problem. The test results of LSTPA, DGEA, SSCEA, IM-MOEA/D, FDV, ATLMOEA, LERD, and MPSLMOCSO are shown in Table 13. Where “–” indicates that the value is meaningless. It can be observed that the proposed algorithm performs exceptionally well across the five problems, demonstrating competitiveness when facing the TREE problem.

**Table 13:** The statistical HV results with LSTPA, DGEA, SSCEA, IM-MOEA/D, FDV, ATLMOEA, LERD and MPSLMOCSO on 2-objective TREE test problems.

Problem	D	LSTPA	DGEA	SSCEA	IM-MOEA/D	FDV	ATLMOEA	LERD	MPSLMOCSO
TREE1	3000	1.3016e−1 (1.67e−1)	7.8779e−1 (4.30e−2)	–	–	7.2976e−1 (3.56e−2)	2.1607e−1 (1.63e−1)	7.7883e−1 (5.65e−3)	**8.1638e−1 (1.24e−3)**
TREE2	3000	8.4210e−2 (2.09e−1)	8.3758e−1 (8.62e−3)	–	–	7.7301e−1 (2.96e−3)	2.9511e−1 (1.18e−1)	7.9759e−1 (5.40e−3)	**8.4207e−1 (5.09e−4)**
TREE3	6000	9.2084e−2 (2.03e−6)	6.7386e−1 (1.87e−1)	–	–	–	1.0955e−1 (5.48e−2)	7.6391e−1 (1.70e−2)	**8.8005e−1 (1.36e−3)**
TREE4	6000	8.6945e−2 (4.05e−5)	5.3655e−1 (4.05e−1)	–	–	–	8.1712e−3 (1.63e−2)	2.0221e−1 (1.48e−1)	**9.2884e−1 (9.26e−3)**
TREE5	6000	1.0650e−1 (9.52e−6)	7.7996e−1 (1.56e−1)	–	–	–	5.2084e−1 (2.69e−1)	7.5762e−1 (2.18e−2)	**8.5966e−1 (5.11e−3)**
+/−/=		0/5/0	0/5/0	0/0/0	0/0/0	0/2/0	0/5/0	0/5/0	

### Computational complexity

The complexity of initializing the population in MPSOF is *O*(*ND*). In the first and second stages, the complexity is mainly determined by the chosen optimization algorithm, variable grouping method and weighted optimization. Taking the LMOCSO algorithm as an example, its algorithmic complexity is * O*((13/4+9/2*M*)*N*^2^ + (3*M*/2 + *D*)*N*). MPSOF uses an ordered grouping method, which has a complexity of *O*(*D*log(*D*)). The complexity of weighted optimization depends on the chosen optimization algorithm. The population is updated using optimized weights in both stages, with complexities of *O*((*N*+*D*)*q*) and *O*((1+*N*+3*D*)*q*), respectively. In the third stage, the population is updated as a whole using the chosen optimization algorithm, and the complexity is consistent with that of the selected algorithm. Therefore, the overall complexity of MPSOF can be translated as *O*((13/4+9/2*M*)*N*^2^ + (3*M*/2 + *D*)*N* + *D*log(*D*) + (*N*+*D*)*q*).

**Figure 9:**
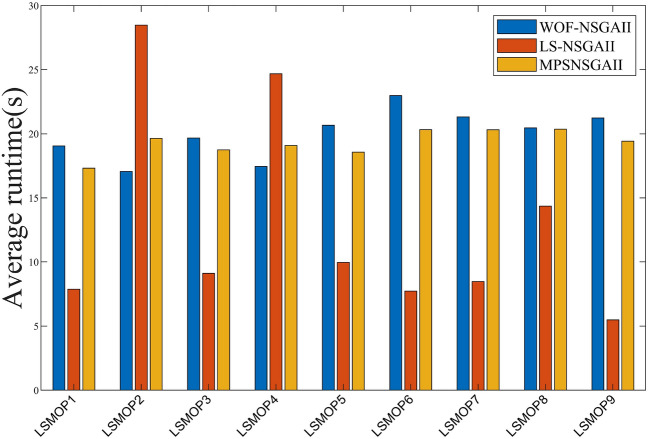
The running time of WOF, LSMOF and MPAOF on NSGAII.

The running times of the three algorithmic frameworks on NSGAII on the second type of experiments are plotted in Fig. 9. It can be observed that LS-NSGAII exhibits some advantages over MPSNSGAII due to its optimization acceleration by combining weights with reference directions. However, the test results in Tables 3 and 4 show that this bidirectional search capability is weak and struggles to comprehensively search the huge solution space. The running time of MPSNSGAII is lower than that of WOF-NSGAII for all problems except for the LSMOP2 and LSMOP4 problems. This is because MPSNSGAII removes duplicate weights during optimization, reducing the duplication of updating individuals and the time spent evaluating duplicate individuals. Overall, MPSOF achieves a good trade-off between efficiency and performance compared to the two algorithmic frameworks.

## Conclusion

This paper introduces a multi-population multi-stage adaptive weighting framework designed specifically for solving LSMOPs. Based on the traditional weighted optimization framework, MPSOF divides the entire optimization process into three stages. Initially, the first stage adopts the traditional weighted update method to maintain a satisfactory convergence rate for the algorithm. Subsequently, the second stage designs an adaptive mixed individual update strategy, enhancing individual diversity and convergence. Lastly, the third stage incorporates a reasonable optimization algorithm to optimize the global variables, thereby improving the accuracy of solutions.

By conducting comparative analysis on the results of three types of experiments, it is verified that MPSOF effectively upgrades the capabilities of the original algorithms and outperforms the traditional algorithmic frameworks. Furthermore, when compared to some advanced optimization algorithms, it still shows good properties on most LSMOPs without using the optimal optimization algorithm as the optimizer.

The experimental findings also demonstrate that MPSOF does not obtain optimal solutions for all problems, which is in line with the “no free lunch principle”. Compared to WOF, the algorithm in this paper adds two parameters $\theta$ and $\delta$ for three-stage switching. This paper uses parameter sensitivity analysis to obtain a compromise solution. In practical problem, the chosen parameters are not optimal and lack adaptivity. Finding a solution for designing adaptive parameters according to problem characteristics and population dynamics in the evolutionary process remains an unresolved challenge for future research.

## Data Availability

All relevant data are within the paper, and the code available from the corresponding author on reasonable request.
